# The dose of behavioral interventions to prevent and treat childhood obesity: a systematic review and meta-regression

**DOI:** 10.1186/s12966-017-0615-7

**Published:** 2017-11-15

**Authors:** William J. Heerman, Meghan M. JaKa, Jerica M. Berge, Erika S. Trapl, Evan C. Sommer, Lauren R. Samuels, Natalie Jackson, Jacob L. Haapala, Alicia S. Kunin-Batson, Barbara A. Olson-Bullis, Heather K. Hardin, Nancy E. Sherwood, Shari L. Barkin

**Affiliations:** 1 Department of Pediatrics, Vanderbilt University Medical Center, 2146 Belcourt Ave, 2nd Floor, Nashville, TN 37212 USA; 2 Division of Applied Research, Allina Health, Minneapolis, USA; 3 Department of Family Medicine and Community Health, University of Minnesota Medical School, Minneapolis, USA; 4 Department of Population and Quantitative Health Sciences, Case Western Reserve University, Cleveland, USA; 5 Division of Epidemiology and Community Health, University of Minnesota, Minneapolis, USA; 6 HealthPartners Institute, Minneapolis, USA; 7 Frances Payne Bolton School of Nursing, Case Western Reserve University, Cleveland, USA; 8 Department of Biostatistics, Vanderbilt University School of Medicine, Nashville, USA

**Keywords:** Dose, Behavioral intervention, Childhood obesity, Health behavior, Systematic review

## Abstract

**Background:**

A better understanding of the optimal “dose” of behavioral interventions to affect change in weight-related outcomes is a critical topic for childhood obesity intervention research. The objective of this review was to quantify the relationship between dose and outcome in behavioral trials targeting childhood obesity to guide future intervention development.

**Methods:**

A systematic review and meta-regression included randomized controlled trials published between 1990 and June 2017 that tested a behavioral intervention for obesity among children 2–18 years old. Searches were conducted among PubMed (Web-based), Cumulative Index to Nursing and Allied Health Literature (EBSCO platform), PsycINFO (Ovid platform) and EMBASE (Ovid Platform). Two coders independently reviewed and abstracted each included study. Dose was extracted as intended intervention duration, number of sessions, and length of sessions. Standardized effect sizes were calculated from change in weight-related outcome (e.g., BMI-Z score).

**Results:**

Of the 258 studies identified, 133 had sufficient data to be included in the meta-regression. Average intended total contact (# sessions x length of sessions) was 27.7 (SD 32.2) hours and average duration was 26.0 (SD 23.4) weeks. When controlling for study covariates, a random-effects meta-regression revealed no significant association between contact hours, intended duration or their interaction and effect size.

**Conclusions:**

This systematic review identified wide variation in the dose of behavioral interventions to prevent and treat pediatric obesity, but was unable to detect a clear relationship between dose and weight-related outcomes. There is insufficient evidence to provide quantitative guidance for future intervention development. One limitation of this review was the ability to uniformly quantify dose due to a wide range of reporting strategies. Future trials should report dose intended, delivered, and received to facilitate quantitative evaluation of optimal dose.

**Trial registrations:**

The protocol was registered on PROSPERO (Registration #CRD42016036124).

**Electronic supplementary material:**

The online version of this article (10.1186/s12966-017-0615-7) contains supplementary material, which is available to authorized users.

## Background

In the last several decades there have been a significant number of behavioral interventions designed to support healthy childhood growth and prevent or treat childhood obesity. Systematic reviews that have attempted to draw meaningful conclusions from randomized controlled trials of these interventions have pointed to modestly efficacious results [1–6]. These same reviews highlight a wide range of variability both in the approach taken (e.g., type of intervention, setting, and age group) and in the evaluative methods employed (e.g., strength of trial designs, measurement approaches, and reporting of processes). The difficulty in synthesizing such a voluminous and varied literature leaves the public health community—researchers, clinicians, policy-makers, and patients—without a clear consensus as to the best approach to implementing both efficacious and sustainable solutions to the childhood obesity epidemic.

Establishing the optimum dose of behavioral interventions to prevent and treat childhood obesity is a critical question for addressing the obesity epidemic [7]. Unlike drug trials, the dose of a behavioral intervention is not easily quantified, and unlike drug trials there is a paucity of data on how the dose of a behavioral intervention is related to its outcome. Available literature from behavioral interventions to reduce adult obesity and its associated complications (i.e., diabetes incidence, glycemic control, hypertension, and hyperlipidemia) suggest that there is an association between higher dose and better efficacy [8]. Furthermore, a recent meta-analysis of 20 studies by Janicke et al. found that the dose of comprehensive behavioral family lifestyle interventions in the community or in outpatient clinical settings was associated with their efficacy at supporting healthy childhood growth [9]. With several areas of research supporting the notion that dose is related to efficacy in behavioral interventions, the pediatric research community is left with a critical unanswered question: how much exposure to a behavioral intervention over what period of time is necessary to affect sustainable behavior change and reduce childhood obesity?

In the context of behavioral obesity interventions dose can be characterized as a function of the following attributes: intervention duration, the number of sessions, and the length of sessions [10–12]. Duration is the time over which the active components of an intervention are implemented, and it can be measured in days, weeks, months, or years. The number of sessions/contacts with participants occurs over the intervention duration (e.g., one session per week over three months, or 12 sessions). The length of sessions refers to the amount of time each contact lasts between the interventionist and the participant and generally is measured in minutes or hours. These dose parameters collectively determine a cumulative amount of intervention or “dose”. Furthermore, an optimal dose can be defined as either the maximally efficacious dose that is not conditional on patient adherence (intended dose) or the maximally effective (actual received or observed) dose that is conditional on adherence [13].

Developing a clear understanding of how the dose of a behavioral intervention is related to the outcome is critical for both causal inference and for developing and disseminating future interventions that support healthy childhood growth. The purpose of this study is to review the existing literature on behavioral interventions to prevent and treat childhood obesity and to use quantitative methods to better understand how dose was related to outcome. Our aims were 1) to describe the distribution of dose (i.e., duration, number of sessions, and length of sessions) in existing behavioral trials for childhood obesity in a range of settings, and then 2) to provide researchers with guidance as future interventions are developed and implemented regarding the minimum dose of a behavioral intervention that would likely be necessary to achieve meaningful and sustainable improvements in childhood obesity. This approach fills the gap in the existing literature by attempting to quantify the “dose” of a behavioral intervention that is needed to affect change in childhood obesity.

## Methods

### Protocol and registration

We conducted a systematic review of the literature using a pre-specified protocol and in accordance with the Preferred Reporting Items for Systematic Reviews and Meta-Analyses (PRISMA) guidelines [14]. The protocol was registered on PROSPERO (Registration #CRD42016036124). The initial literature search was completed in March 2014, with an update in June 2017.

### Information sources and search strategy

The full methods of the search strategy used in this systematic review have been previously published, and complete search terms and parameters are available on PROSPERO [11]. Briefly, the PubMed (Web-based), Cumulative Index to Nursing and Allied Health Literature (EBSCO platform), PsycINFO (Ovid platform) and EMBASE (Ovid Platform) databases were searched by a trained health sciences librarian to identify randomized controlled trials (RCTs) or intervention studies on the treatment or prevention of childhood obesity. To search PubMed we used the medical subject headings (MeSH) to define the concepts of obesity, overweight or body mass index; treatment, therapy, or prevention; children, childhood, adolescents or pediatric (under 18 years of age); and RCTs or intervention studies.

To augment this search strategy, bibliographies from selected key systematic reviews were scanned to identify additional publications.

### Study selection process

A team of eight coders participated in a two-step selection process. First, the title and abstract of each identified article was independently reviewed by two coders to make initial exclusions. For the remaining articles, two coders independently read the full text to determine final inclusion or exclusion. The lead reviewer adjudicated discrepancies during both steps of the process, involving the larger group of coders when necessary. The initial literature search resulted in 4692 articles, and the updated literature search resulted in 876 additional articles. The updated literature search also identified 10 new articles associated with previously included studies, 6 of which resulted in updating data in the analysis.

Studies were included if they were 1) randomized controlled trials, 2) tested a behavior change intervention to treat or prevent obesity (i.e., focused on healthy behaviors like diet, physical activity, sleep or media use without meeting exclusion criteria below), 3) included children between ages 2 and 18 years old at the time of randomization, 4) had sufficient data to calculate the main analytic variables of dose and a weight-related outcome (e.g., BMI-Z score), and 5) were published in English between January 1990 and June 2017. Studies were excluded if they included 1) an intervention targeted at pregnant women, 2) children who were underweight, 3) children who were hospitalized, in residential overnight camps, or in assisted living, 4) pharmacologic or surgical interventions, 5) prescribed diet or exercise interventions without a behavior change component, or 6) interventions delivered only during the school day or community-wide interventions (where individual dose was not measured). If a trial contained more than one intervention arm, only one was selected for review based on how many of the following features it had: 1) in person or individually delivered, 2) enhanced or multicomponent, (i.e., elements added to the basic intervention being tested), 3) parent and child participants. Because the unit of analysis for this approach is the study, if multiple articles were identified for a single study, then all identified articles were used to extract data for any given study [11].

### Data extraction process and elements

Two coders independently reviewed all included studies to determine study characteristics. For each study several characteristics about the study were collected, including study year, participant age (coded: only 2–5 or 6–11 or both, only 12–14 or 15–18, or both, and all other combinations of 2–5, 6–11, 12–14, and 15–18), intervention mode (coded: in person only, and combination of in person and either phone, printed material, computer/app/video game, email/text, or other), intervention setting (coded: clinic or university only, school or community only, and all other combinations of home, school, clinic, community, university, environmental, or other), intervention format (coded: individual only, and group only, or individual + group), and intervention participants (coded: child only, and parent only, or parent + child, or parent + child + other family). The Delphi checklist was used to measure study quality, which includes nine items that assess various aspects of study quality with a yes or no response [15]. By virtue of the inclusion/exclusion criteria several of these criteria were necessarily met, consequently we included only whether studies used an intention to treat analysis or not (coded not intention to treat, and intention to treat) in our analytic model (described below). Decisions regarding coding of covariates were initially based on theory and revised based on the uniformity of reporting across the included studies.

#### Data elements

##### Dose variables

A single coder conducted subsequent extraction of quantitative dose and outcome components with 20% of the studies reviewed by a second coder (>95% concordance). Three components of dose were extracted: duration of intervention, the number of in person sessions, and the length of sessions in hours. The primary exposure variable of total in person contact hours was then calculated from the product of number of sessions and length of sessions. For example, a study with 12 weekly, 60-min sessions over 6 months would have the following parameters: 12 contact hours with a duration of 6 months. Of the 258 studies identified by the systematic review, 193 had complete data on at least one in-person dose component. Because only 28 studies had data on the dose of a phone component and only 40 studies had data on the dose of print materials, these did not factor into the numerical calculation of dose. We included data only on the intended dose and not the dose received, as the vast majority of studies did not report sufficient quantitative measures of dose received. For the 41 studies where it was possible to compare the dose intended with the dose received, the intended-received correlation was 0.95 (*p* < 0.001). This correlation suggests that the reported dose received was highly related to the reported dose intended, noting the potential bias where studies of lower quality or studies that suffered from lower rates of fidelity to the intended dose may not have reported the dose received as frequently as higher quality studies.

##### BMI outcome variables

Because dose is typically only delivered to the intervention participants, the primary effect size was derived from change in weight-related outcome in the intervention group only. In studies where multiple follow-up times were reported, we used the first follow-up with complete data. Furthermore, there was significant variability in control or comparator arms in these studies, making a quantitative comparison between the primary intervention and comparator infeasible. Because different types of outcomes were reported in the literature, all weight-related measurements were recorded for each study, most commonly weight, body mass index (BMI), BMI Z-score, and % over BMI. The standardized mean effect size was calculated to allow comparability across the different types of weight-related outcomes. However, non-standardized weight outcomes are highly sensitive to individual participant age. Therefore, when multiple outcome types were available, we prioritized hierarchically as follows: BMI-Z, BMI percentile, % over BMI, weight, followed by raw BMI. We also used meta-regression to test whether effect size magnitude was related to the type of outcome used with each type of outcome dummy coded and compared to BMI-Z score as the reference group. This process demonstrated that the distribution of effect sizes from raw BMI were significantly different than those calculated from BMI-Z scores. Therefore, studies that reported raw BMI only were not analyzed (*N* = 27), and coded as missing the outcome. None of the other effect size types were significantly different from BMI-Z score.

### Statistical analysis

Because we were interested in the effect of the intervention dose on pre-post differences within the intervention group, effect sizes were calculated for each study using the standardized mean pre-post difference methodology [16]. In this context, the formula for the sample estimate of the standardized mean difference (*d*) is.

$$ d=\frac{M_{\mathrm{diff}}}{SD_{\mathrm{within}}}=\frac{M_{\mathrm{post}}-{M}_{\mathrm{pre}}}{SD_{\mathrm{within}}}, $$

where *M*_diff_ is the mean outcome change or the mean difference between pre and post outcome in the intervention group. *M*_pre_ and *M*_post_ are the mean outcome at baseline and follow up, respectively, and *SD*_within_ is the sample standard deviation between time periods (i.e., pre-post). If *SD*_within_ was not reported, then it was imputed from the pre-post BMI correlation and either the standard deviation of the outcome differences (*SD*_diff_) when possible or from the pre and post standard deviations *(SD*_pre_ and *SD*_post_, respectively) when *SD*_diff_ was not reported. Because *SD*_pre_ and *SD*_post_ were correlated at 0.94 (*p* < 0.001), when only one of *SD*_pre_ or *SD*_post_ was available, the SD that was present was imputed for the one that was missing [17]. Finally, when it was not possible to calculate the pre-post BMI correlation for a study (which was necessary to impute the SD of BMI for the effect size calculation), the following schema was used to estimate it: *r* = 0.90 for studies 26 weeks or shorter; *r* = 0.80 for studies 52 weeks or shorter, but longer than 26 weeks; and *r* = 0.70 for studies longer than 52 weeks.

We conducted a random-effects meta-regression using inverse variance weighting to characterize the relationship between the total in-person contact hours, total duration and intervention group effect size [18]. The unit of analysis was individual study. Random-effects modeling was used to allow random error to occur at both the subject and study levels. Random-effects modeling is generally preferred when it is expected that the effect sizes will be heterogeneous or when it is desired to generalize the results beyond the subsample of analyzed studies [19]. All effect sizes were calculated so that a negative value indicated that the outcome decreased at follow up when compared to baseline, while a positive effect size indicates that outcome increased over the course of the study. Cochrane’s *Q* and the *I* ^2^ index were used to examine the degree of effect size heterogeneity.

The base model evaluating the relationship between dose and effect size contained three independent variables: mean-centered contact hours, mean-centered intervention duration, and the interaction of centered contact hours and centered intervention duration. The interaction was included to account for the possibility that the relationship between contact hours and effect size may depend on study duration. For example, the effect of contact hours in a study with 40 contact hours over 6 months might be expected to be different than the effect of contact hours in a study with 40 contact hours over 2 years. The full model controlled for additional covariates that were theorized to be confounders of the relationship between dose variables and change in the outcome. These covariates were coded as follows: 1) study year (to account for temporal trends in efficacy of studies; coded continuously) 2) intention to treat (Yes vs. No), 3) participant age group (12–18 years vs. 2–11 years), 4) intervention mode (multi-modality vs. in person only), 5) setting (school/community vs. multi-location vs. clinic/university), 6) intervention format (group vs. individual/group or individual only), 7) intervention participants (parent or parent/child vs. child only), and 8) baseline weight status of study participants (normal + overweight or obese vs. overweight or obese only). We used the Delphi criteria to code for study quality, and included intention to treat as a surrogate for study quality in our analytic model. We also included baseline weight status as a covariate to control for studies primarily aimed at obesity treatment. Finally, separate stratified meta-regressions were run on studies that had specific characteristics to explore for possible dose effects specific to different types of interventions (Additional file 1: Table S2). This additional analysis using stratified meta-regressions should be interpreted with caution due to potential bias and power issues related to sample restriction, but was done for exploratory purposes. All data were extracted into a secure REDCap database and subsequent analyses were conducted using Stata version 14.2 and R version 3.4.0 [20, 21].

Following recommendations in the Cochrane Handbook for Systematic Reviews of Interventions [17], we created a funnel plot to visually assess whether bias from small-study effects may have been present. Additionally, because visual inspection is largely subjective, we conducted the regression test for funnel-plot asymmetry proposed by Egger et al. [22] To further evaluate the influence of small-study effects, we then compared the fixed- and random-effects estimates of the overall effect size. To remove bias that can occur when estimating effect size in studies with small sample sizes, the standardized mean difference effect size, *d*, was converted to Hedges’ *g* via a correction factor, *J* [16].

## Results

**Fig. 1:**
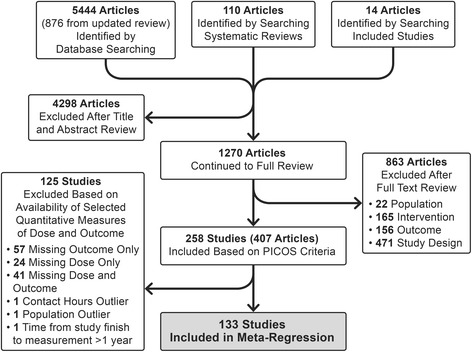
Flow Diagram

**Fig. 2:**
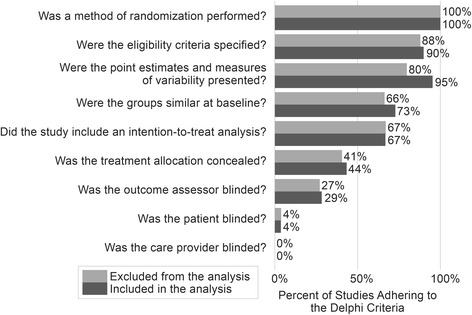
Assessment of Study Quality using the Delphi Criteria. The percent of studies adhering to the Delphi criteria is shown, comparing studies included in the analytic sample vs. studies excluded from the analytic sample. It is important to note that if a study did not specifically report adherence, then the study would be coded as non-adherent

The literature search returned 1270 articles for full review, 863 of which were excluded (471 for study design, 165 for intervention type, 156 for outcome measure, and 22 for population studied), resulting in 258 studies that were abstracted. Of the 258 studies identified by the systematic review, 125 were excluded from the meta-regression. We excluded 123 studies because of incomplete data on effect size, dose, or both. We excluded two additional studies: one study because its initial follow-up time point was one year after the study was completed, and one study because its target population differed greatly from the target populations of the other included studies. This led to the final analytic sample of 133 studies that had acceptable data on dose and a calculable effect size (Fig. 1). See the supplementary material (Additional file 1: Table S1) for full list of reviewed studies. Using the Delphi Criteria, there were no differences in study quality (i.e., internal validity) between the studies included vs. excluded in the analytic sample (Fig. 2).

The distribution of dose components is shown in Table 1. The average intended total contact hours (# sessions x length of sessions) of the 133 studies included was 27.7 (SD 32.2) hours, and the median was 18 (IQR 10, 36) hours. Based on previously published cut-points, 23% of the included studies were categorized as low intensity (<10 h), 49% were categorized as medium intensity (≥10 h & <36 h), and 28% were categorized as high intensity (≥ 36 h) [8]. The average intervention duration for included studies was 26.0 (SD 23.4) weeks and the median intervention duration was 17 (IQR 12, 26) weeks. The intervention duration was as follows: 37% of studies were <3 months, 41% were ≥3 and <6 months, 5% were ≥6 and <9 months, and 17% were ≥9 months.

**Table 1:** Descriptive Statistics of Included and Excluded Studies

	Analytic Sample (*n* = 133)		Excluded Sample (*n* = 125)	
	Median or Frequency	IQR or Percent	Median or Frequency	IQR or Percent
Hedges’ *g* effect size^a^	−0.25	[−0.83, 0.33]	−0.40^b^	[−1.09, 0.29]
Contact hours	18.0	[10.0, 36.0]	12.5^c^	[7.6, 27.0]
Duration (in weeks)	17.3	[12.0, 26.0]	25.5^d^	[12.0, 35.0]
Study Year	2010	[2012, 2014]	2012	[2008, 2014]
Intention to treat				
Yes	89	67%	84	67%
No	44	33%	41	33%
Participant age group				
2–11 only	52	39%	49	39%
12–18 only	22	17%	23	18%
Other combination	58	44%	53	42%
Intervention mode				
In-person only	57	43%	42	34%
In-person plus other	76	57%	65	52%
No in-person dose	0	0%	18	14%
Setting^e^				
School/community only	27	22%	34	29%
Clinic/university only	55	46%	26	22%
All other combinations	38	32%	59	50%
Format^f^				
Individual only	26	20%	56	45%
Group only or both	106	80%	68	55%
Participants				
Child only	24	18%	28	22%
Parent only, or parent and child, or parent and child and other family	109	82%	97	78%
Weight Status				
Normal weight and overweight or obese	28	21%	45	36%
Overweight or obese only	105	79%	77	62%
Normal weight only	0	0%	3	2%

^a^The pooled effect size was estimated using the DerSimonian and Laird methodology for random-effects meta-analysis. The variance of the effect sizes is described by the interval from ±2*τ around the random-effects pooled estimate, which is an approximate 95% range of the effect sizes

^b^Effect size was missing in 98 of the excluded studies (*n* = 27)

^c^Contact hours was missing in 63 of the excluded studies (*n* = 62)

^d^Duration was missing in 25 of the excluded studies (*n* = 100)

^e^Setting was missing in 13 of the analyzed studies (*n* = 120) and 6 of the excluded studies (*n* = 119)

^f^Format was missing for 1 of the analyzed studies (*n* = 132) and 1 of the excluded studies (*n* = 124)

There was significant variability of the effect sizes (Additional file 1: Figure S1). Based on the results of a random effects meta-analysis, heterogeneity was significant (χ [2] = 4868; d. f. = 132; *p* < 0.001) with 97.3% of the variation in effect size attributable to heterogeneity (*I* ^2^). The estimate of between-study variance was 0.08 (τ [2]). At the 95% confidence level, 75 studies (56%) demonstrated a statistically significant decrease in the standardized outcome (e.g., BMI-Z), 53 studies (40%) demonstrated no significant change, and 5 studies (4%) demonstrated a significant increase in the standardized outcome.

**Fig. 3:**
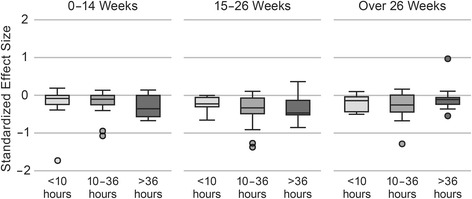
Box Plot of Standardized Effect Size by Dose Components. For each category of duration (0–14 weeks, 15–26 weeks, over 26 weeks) the standardized effect sizes are shown for each category of contact hours (<10 h, 10–36 h, over 36 h). There does not appear to be a trend by duration or contact hours that would suggest an association between either component and effect size. Note that this approach does not take into account the weighting of studies that is possible through meta-regression techniques. A negative standardized effect size represents a decrease in weight-related outcome and a positive standardized effect size represents an increase in weight-related outcome

In the base model that examined the relationship between intended dose and effect size, there was no detectable relationship between standardized effect size and the total contact hours (Β = −0.001; 95% CI [−0.003, 0.002]; *p* = 0.5), the duration of the intervention (Β = 0.0003; 95% CI [−0.002, 0.003]; *p* = 0.8) or their interaction (Β = 0.00005; 95% CI [−0.000008, 0.0001]; *p* = 0.09). When treating dose categorically, there was no apparent trend toward an association between any component of dose and the effect size (Fig. 3). In the full model that accounted for both dose and additional study characteristics, there was not a statistically significant association between contact hours, duration or their interaction and effect size (Table 2). When evaluating for any association between dose and effect size in certain types of studies (i.e., stratified analyses), we found a very small but statistically significant association between longer studies and worse outcomes when studies recruited normal weight individuals in addition to overweight/obese individuals (Additional file 1: Table S2).

**Table 2:** Meta-regression comparing the dose of the interventions with the standardized effect size, controlling for study characteristics

*n* = 119	Coefficient	95% CI		*p*-value
Contact Hours (centered)	0.000	−0.003,	0.002	0.79
Duration (centered)	0.001	−0.002,	0.003	0.44
Contact Hours x Duration	0.000	0.000,	0.000	0.29
Article Year	−0.007	−0.025,	0.011	0.46
Intention to Treat				
No	–	–		–
Yes	−0.004	−0.137,	0.129	0.95
Age				
2–11 years	–	–		–
12–18 years	0.123	−0.044,	0.290	0.15
Combination	0.032	−0.098,	0.161	0.63
Mode				
In Person Only	–	–		–
In Person + Other Mode	0.040	−0.087,	0.166	0.53
Setting				
Clinic or University	–	–		–
School or Community	0.107	−0.053,	0.267	0.19
Combination	0.021	−0.117,	0.159	0.76
Format				
Individual Only	–	–		–
Group or Individual + Group	−0.106	−0.258,	0.045	0.17
Participants				
Child Only	–	–		–
Parent or Parent + Child	−0.083	−0.239,	0.073	0.30
Weight Status				
Normal + Overweight or Obese	–	–		–
Overweight or Obese Only	−0.277	−0.425,	−0.130	<0.001

*N* = 119 with complete data on all covariates

**Fig. 4:**
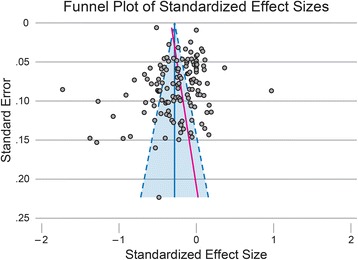
Funnel Plot of Standardized Effect Sizes. The funnel plot demonstrates minimal visual asymmetry, in the direction of excess small studies that resulted in a “negative” outcome (i.e., weight gain = positive effect size). This is supported by the estimated bias coefficient from the Egger test (bias = 1.6, *p* = 0.03, shown as red solid line). Dotted lines represent the pseudo-95% confidence interval

The funnel plot (Fig. 4) demonstrated minimal visual asymmetry. This was supported by the estimated bias coefficient from the Egger test (bias = 1.6, *p* = 0.03). To further evaluate for potential small-study bias, we compared the estimate from the random effects model used in Table 1 with that from a fixed-effects model (in which, relative to the random effects model, smaller studies are given smaller weights and larger studies are given larger weights). The fixed effects model yielded an estimate of −0.28 (*p* < 0.001), which is very similar to the random effects estimate (−0.25, *p* < 0.001). Taken together, these results suggest that the meta-regression estimates are not biased from the lack of inclusion of small studies with “negative findings” (i.e., weight gain).

## Discussion

This systematic review identified wide variation in the dose of behavioral interventions to prevent and treat pediatric obesity. In addition, there was no association between dose and changes in weight-related outcomes. Based on the results of these analyses we are unable to provide guidance to the field regarding a minimally effective dose that should be targeted as new behavioral interventions are developed and implemented to treat or prevent childhood obesity. We hypothesize that one of the main reasons we did not find an association between treatment intensity and effect size is that behavior change occurs at a different threshold of exposure to the intervention for each person. Thus, for some people a low “dose” will result in rapid behavior change, but for others an extensive “dose” will be required to even begin the process of behavior change. This level of detail could not be explored using meta-regression techniques, as we did not have individual-level data. Future research should consider focusing on non-linear modeling of dose components as they relate to change in weight-related outcomes. It may have also been that many of the studies had inadequate intervention content/behavioral change strategies. Finally, an individual’s healthy behaviors are the product of multiple levels of influence, including family, environment, communities, and local policies [23]. The current literature does not often report on the complex environmental determinants of a person’s behavior, which makes knowing how to apply these results in a specific context challenging, though this would be an important future direction to consider.

Our study adds to the literature on how the dose of a behavioral intervention is related to pediatric obesity outcomes, however the existing literature provides limited context for these findings. In one previous meta-analysis of 20 behavioral interventions to treat pediatric obesity in community/outpatient settings, dose was found to be a moderator of the relationship between study and outcome [9]. Several systematic reviews have been unable to assess dose or have pointed to the challenges associated with measuring dose appropriately in these contexts [1, 2, 24, 25]. In adult behavior change literature, there has been some preliminary indication that more intensive (i.e., more contact hours) interventions are related to a higher degree of reduction in cardiovascular disease risk [8]. In addition, the smoking cessation literature has demonstrated a dose-response relationship for behavioral interventions in clinic, where more intensive interventions are both better received and more effective at achieving smoking cessation [26, 27]. But these same relationships between dose and outcome in behavioral trials have also been challenging to characterize in other fields, including medication adherence in chronic medication conditions and HIV-related research [28–30]. Taken together, these bodies of literature suggest that while there may be a relationship between the dose of behavior-change interventions and health behaviors, understanding how the dose of a behavioral intervention is related to the target outcome is challenging in any behavior-change context.

The reasons why a relationship was not found between dose and effect size in the current analysis is unclear. We found that there were several limitations to conducting the analysis as we had planned. First, there was wide variation in reporting on the dose of an intervention, making it challenging to appropriately and uniformly quantify the dose delivered and/or received in the intervention. While the underlying characteristics of the studies that were included and excluded based on availability of a dose or outcome measure were not substantially different from each other, we cannot exclude a potential selection bias, which would likely bias the results to the null. Furthermore, many studies did not include sufficient information on multi-modal components (e.g., phone calls, web-based material) to include in a quantitative evaluation of dose of all intervention components. While the multi-modal nature of interventions was not associated with the effect size in the adjusted meta-regression, we could have misclassified the primary exposure for multi-modal studies, which again may have biased the results to the null. The testing of novel trial designs, including the multi-phasic optimization strategy (MOST) and sequential multiple assignment randomized trials (SMART) may be particularly well suited to characterizing these relationships between dose and outcome [31]. We examined the potential for small-study or publication bias, which did not appear to exert a significant effect. The potential for bias from study quality was partially mitigated by the RCT inclusion criterion and by controlling for intent-to-treat study design. However, we primarily used measures of internal validity to measure study quality. As research in the field of behavioral obesity moves from an efficacy approach (where these results are most applicable) to a dissemination and implementation framework, future attempts at systematic reviews should evaluate studies based on external validity (i.e., RE-AIM framework) [32–34] to help guide translation of results into communities. An additional limitation to this study is that we may not have identified all studies relevant to the literature review, excluding relevant findings that could have influenced results. Finally, we could not report on the dose actually received by participants because it was not reported consistently across the literature.

## Conclusions

In conclusion, the available literature does not provide sufficient evidence to suggest a minimum behavioral dose to prevent or treat childhood obesity. It is not clear, whether the lack of an association between dose intensity and change in weight-related outcome is because behavior change is non-linear or whether variability in the reporting of both dose and outcome obscured a relationship. Careful attention to this in large ongoing randomized trials may be an effective approach to understand the complex issue of dose in behavioral interventions. Consistent with ongoing work from the Template for Intervention Description and Replication (TIDieR) and NIH Behavior Change consortium, future researchers should quantitatively identify dose intended, dose delivered, and dose received across each intervention format (individual, group, online, text, phone call, mailing) in addition to effect size when reporting results of childhood weight management trials to facilitate quantitative evaluation of how dose is associated with behavior change [35].

## Additional file

Additional file 1:
**Figure S1.** Forest plot demonstrating a visual representation of the variability in standardized effect size. **Table S1.** Dose of behavioral intervention and intervention characteristics by study and a full reference list of all studies reviewed. Table provides details of all articles reviewed in this meta-regression. **Table S2.** Meta-regression evaluating the relationship between dose and standardized effect size stratified by study characteristics. (PDF 1938 kb)
