# Supplementary material for: The dose of behavioral interventions to prevent and treat childhood obesity: a systematic review and meta-regression
Source: Int J Behav Nutr Phys Act. 2017 Nov 15;14:157. doi: 10.1186/s12966-017-0615-7 (PMC5688650; doi:10.1186/s12966-017-0615-7)
Supplement: Additional file 1: — Figure S1. Forest plot demonstrating a visual representation of the variability in standardized effect size. Table S1. Dose of behavioral intervention and intervention characteristics by study and a full reference list of all studies reviewed. Table provides details of all articles reviewed in this meta-regression. Table S2. Meta-regression evaluating the relationship between dose and standardized effect size stratified by study characteristics. (PDF 1938 kb) [file 12966_2017_615_MOESM1_ESM.pdf]

Appendix (Including Figure S1, Table S1, Table S2, and complete reference list):

Figure S1: Forest plot demonstrating the variability in effect sizes for the 133 studies included in the meta-regression. The pooled effect size is not reported, as it would have represented a comparison of the intervention and control groups of these studies, instead of the pre-post meta-regression that this study reports. Consequently, no conclusion about the overall efficacy of these trials should be made from this plot. A negative standardized effect size represents a decrease in weight-related outcome in the intervention group and a positive standardized effect size represents an increase in weight-related in the intervention group. The studies are sorted by magnitude of effect size, but can be referenced by study ID number in Table S1.

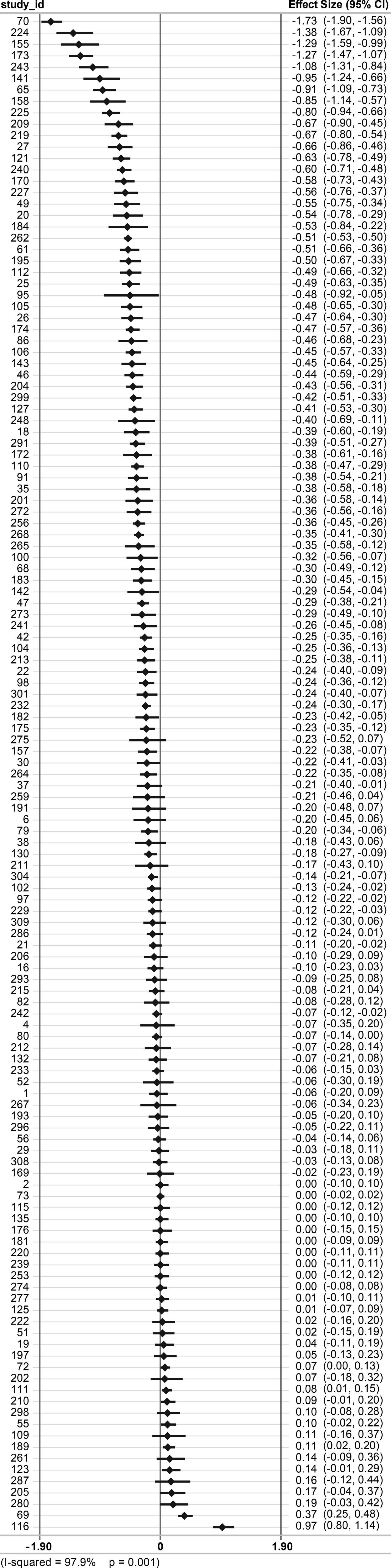
study_id
Effect Size (95% CI)
70 -1.73 (-1.90, -1.56)
224 -1.38 (-1.67, -1.09)
155 -1.29 (-1.59, -0.99)
173 -1.27 (-1.47, -1.07)
243 -1.08 (-1.31, -0.84)
141 -0.95 (-1.24, -0.66)
65 -0.91 (-1.09, -0.73)
158 -0.85 (-1.14, -0.57)
225 -0.80 (-0.94, -0.66)
209 -0.67 (-0.90, -0.45)
219 -0.67 (-0.80, -0.54)
27 -0.66 (-0.86, -0.46)
121 -0.63 (-0.78, -0.49)
240 -0.60 (-0.71, -0.48)
170 -0.58 (-0.73, -0.43)
227 -0.56 (-0.76, -0.37)
49 -0.55 (-0.75, -0.34)
20 -0.54 (-0.78, -0.29)
184 -0.53 (-0.84, -0.22)
262 -0.51 (-0.53, -0.50)
61 -0.51 (-0.66, -0.36)
195 -0.50 (-0.67, -0.33)
112 -0.49 (-0.66, -0.32)
25 -0.49 (-0.63, -0.35)
95 -0.48 (-0.92, -0.05)
105 -0.48 (-0.65, -0.30)
26 -0.47 (-0.64, -0.30)
174 -0.47 (-0.57, -0.36)
86 -0.46 (-0.68, -0.23)
106 -0.45 (-0.57, -0.33)
143 -0.45 (-0.64, -0.25)
46 -0.44 (-0.59, -0.29)
204 -0.43 (-0.56, -0.31)
299 -0.42 (-0.51, -0.33)
127 -0.41 (-0.53, -0.30)
248 -0.40 (-0.69, -0.11)
18 -0.39 (-0.60, -0.19)
291 -0.39 (-0.51, -0.27)
172 -0.38 (-0.61, -0.16)
110 -0.38 (-0.47, -0.29)
91 -0.38 (-0.54, -0.21)
35 -0.38 (-0.58, -0.18)
201 -0.36 (-0.58, -0.14)
272 -0.36 (-0.56, -0.16)
256 -0.36 (-0.45, -0.26)
268 -0.35 (-0.41, -0.30)
265 -0.35 (-0.58, -0.12)
100 -0.32 (-0.56, -0.07)
68 -0.30 (-0.49, -0.12)
183 -0.30 (-0.45, -0.15)
142 -0.29 (-0.54, -0.04)
47 -0.29 (-0.38, -0.21)
273 -0.29 (-0.49, -0.10)
241 -0.26 (-0.45, -0.08)
42 -0.25 (-0.35, -0.16)
104 -0.25 (-0.36, -0.13)
213 -0.25 (-0.38, -0.11)
22 -0.24 (-0.40, -0.09)
98 -0.24 (-0.36, -0.12)
301 -0.24 (-0.40, -0.07)
232 -0.24 (-0.30, -0.17)
182 -0.23 (-0.42, -0.05)
175 -0.23 (-0.35, -0.12)
275 -0.23 (-0.52, 0.07)
157 -0.22 (-0.38, -0.07)
30 -0.22 (-0.41, -0.03)
264 -0.22 (-0.35, -0.08)
37 -0.21 (-0.40, -0.01)
259 -0.21 (-0.46, 0.04)
191 -0.20 (-0.48, 0.07)
6 -0.20 (-0.45, 0.06)
79 -0.20 (-0.34, -0.06)
38 -0.18 (-0.43, 0.06)
130 -0.18 (-0.27, -0.09)
211 -0.17 (-0.43, 0.10)
304 -0.14 (-0.21, -0.07)
102 -0.13 (-0.24, -0.02)
97 -0.12 (-0.22, -0.02)
229 -0.12 (-0.22, -0.03)
309 -0.12 (-0.30, 0.06)
286 -0.12 (-0.24, 0.01)
21 -0.11 (-0.20, -0.02)
206 -0.10 (-0.29, 0.09)
16 -0.10 (-0.23, 0.03)
293 -0.09 (-0.25, 0.08)
215 -0.08 (-0.21, 0.04)
82 -0.08 (-0.28, 0.12)
242 -0.07 (-0.12, -0.02)
4 -0.07 (-0.35, 0.20)
80 -0.07 (-0.14, 0.00)
212 -0.07 (-0.28, 0.14)
132 -0.07 (-0.21, 0.08)
233 -0.06 (-0.15, 0.03)
52 -0.06 (-0.30, 0.19)
1 -0.06 (-0.20, 0.09)
267 -0.06 (-0.34, 0.23)
193 -0.05 (-0.20, 0.10)
296 -0.05 (-0.22, 0.11)
56 -0.04 (-0.14, 0.06)
29 -0.03 (-0.18, 0.11)
308 -0.03 (-0.13, 0.08)
169 -0.02 (-0.23, 0.19)
2 0.00 (-0.10, 0.10)
73 0.00 (-0.02, 0.02)
115 0.00 (-0.12, 0.12)
135 0.00 (-0.10, 0.10)
176 0.00 (-0.15, 0.15)
181 0.00 (-0.09, 0.09)
220 0.00 (-0.11, 0.11)
239 0.00 (-0.11, 0.11)
253 0.00 (-0.12, 0.12)
274 0.00 (-0.08, 0.08)
277 0.01 (-0.10, 0.11)
125 0.01 (-0.07, 0.09)
222 0.02 (-0.16, 0.20)
51 0.02 (-0.15, 0.19)
19 0.04 (-0.11, 0.19)
197 0.05 (-0.13, 0.23)
72 0.07 (0.00, 0.13)
202 0.07 (-0.18, 0.32)
111 0.08 (0.01, 0.15)
210 0.09 (-0.01, 0.20)
298 0.10 (-0.08, 0.28)
55 0.10 (-0.02, 0.22)
109 0.11 (-0.16, 0.37)
189 0.11 (0.02, 0.20)
261 0.14 (-0.09, 0.36)
123 0.14 (-0.01, 0.29)
287 0.16 (-0.12, 0.44)
205 0.17 (-0.04, 0.37)
280 0.19 (-0.03, 0.42)
69 0.37 (0.25, 0.48)
116 0.97 (0.80, 1.14)
-1.90
0
1.90
(I-squared = 97.9% p = 0.001)

Table S1. Dose of behavioral intervention and intervention characteristics by study.

	Study No.	Included in Regression	Intervention Duration (weeks)	No. of Sessions	Length of Sessions (min)	Standardized Effect Size (g)	Intention to Treat	Age	Mode	Setting	Format	Participants
Akdemir (2017)[1]	230	No	21.6	5	40		Yes	Other	In-per only	Sch/com only	Group only or both	P; or P/C; or P/C/O
Andrade (2014)[2-4]	245	No	73.1	13	90		Yes	12-18 only	In-per only	Sch/com only	Group only or both	P; or P/C; or P/C/O
Andre (2015)[5]	248	Yes	8	8	240	-0.402	No	12-18 only	In-per only	Sch/com only	individual only	Child only
Arauz Boudreau (2013)[6]	1	Yes	19	12	90	-0.057	No	Other	In-per & O	All combos	Group only or both	P; or P/C; or P/C/O
Ayala (2010)[7,8]	2	Yes	30.1	7	90	0.000	Yes	2-11 only	In-per & O	All combos	individual only	P; or P/C; or P/C/O
Balagopal (2003)[9-14]	4	Yes	13	39	45	-0.072	No	12-18 only	In-per only	Clin/uni only	individual only	P; or P/C; or P/C/O
Ball (2011)[15]	6	Yes	18	16	52.5	-0.199	Yes		In-per only	Clin/uni only		P; or P/C; or P/C/O
BaniSalameh (2017)[16]	232	Yes	12	6	45	-0.236	No	12-18 only	In-per & O	Sch/com only	Group only or both	P; or P/C; or P/C/O
Banks (2012)[17,18]	7	No		5	87.5		Yes	Other	In-per only	Clin/uni only	individual only	P; or P/C; or P/C/O
Baranowski (2003)[19,20]	9	No	4	20	570		Yes	2-11 only	In-per & O	All combos	Group only or both	P; or P/C; or P/C/O
Baranowski (2011)[21]	10	No					Yes	Other		All combos	individual only	Child only
Barbeau (2007)[22]	11	No	43.3	182	110		Yes	Other	In-per only	Sch/com only	Group only or both	Child only
Barkin (2011)[23]	12	No	26	6	60		Yes	2-11 only	In-per only	All combos	Group only or both	P; or P/C; or P/C/O
Barnes (2015)[24]	233	Yes	8	8	115	-0.061	Yes	Other	In-per only	Sch/com only	Group only or both	P; or P/C; or P/C/O
Bathrellou (2010)[25,26]	13	No	78	20	60		No	Other	In-per only	Clin/uni only	individual only	P; or P/C; or P/C/O
Bean (2012)[27,28,29]	16	Yes	12	12	90	-0.102	Yes	2-11 only	In-per & O	Clin/uni only	Group only or both	P; or P/C; or P/C/O
Beech (2003)[30]	17	No	12	12	90		No	2-11 only	In-per & O	Sch/com only	Group only or both	P; or P/C; or P/C/O
Benestad (2017)[31]	234	No	104	3	240		Yes	Other	In-per & O	Clin/uni only	Group only or both	P; or P/C; or P/C/O
Berkowitz (2013)[32]	18	Yes	52	23	45	-0.395	Yes	12-18 only	In-per & O	All combos	Group only or both	P; or P/C; or P/C/O
Berntsen (2010)[33]	19	Yes	21.6	86	60	0.039	Yes	Other	In-per only	Clin/uni only	Group only or both	P; or P/C; or P/C/O
Berry (2011)[34]	20	Yes	26	15	60	-0.538	No	2-11 only	In-per only	Sch/com only	Group only or both	P; or P/C; or P/C/O
Berry (2014)[35,36]	21	Yes	52	21	105	-0.110	Yes	2-11 only	In-per & O	All combos	Group only	P; or P/C;

**Table S1.** Dose of behavioral intervention and intervention characteristics by study.												
	**Study No.**	**Included in Regression**	**Intervention Duration (weeks)**	**No. of Sessions**	**Length of Sessions (min)**	**Standardized Effect Size (g)**	**Intention to Treat**	**Age**	**Mode**	**Setting**	**Format**	**Participants**
											or both	or P/C/O
Birken (2012)[37]	22	Yes	1	1	10	-0.241	Yes	2-11 only	In-per & O	Clin/uni only	individual only	P; or P/C; or P/C/O
Black (2010)[38]	23	No	47.6	12		-0.025	Yes	Other	In-per & O	All combos	individual only	Child only
Bloom (2013)[39]	24	No	6	6	60		No	Other	In-per only	Clin/uni only	Group only or both	P; or P/C; or P/C/O
Bocca (2012)[40-42]	25	Yes	16	25	72	-0.488	Yes	2-11 only	In-per only	Clin/uni only	Group only or both	P; or P/C; or P/C/O
Bohlin (2017)[43,44]	236	No					Yes	Other	In-per & O	All combos	individual only	P; or P/C; or P/C/O
Bonham (2017)[45,46,47]	237	No	13	12			Yes	12-18 only	In-per only	All combos	individual only	Child only
Boodai (2014)[48]	239	Yes	24	6	60	0.000	Yes	Other	In-per only	Clin/uni only	Group only or both	P; or P/C; or P/C/O
Boutelle (2011)[49]	26	Yes	21.6	21	120	-0.471	No	Other	In-per only	Clin/uni only	Group only or both	P; or P/C; or P/C/O
Boutelle (2013)[50]	27	Yes	21.6	11	20	-0.656	Yes	Other	In-per & O	Clin/uni only	individual only	P; or P/C; or P/C/O
Boutelle (2014)[51]	241	Yes	17.2	28	45	-0.264	Yes	Other	In-per & O	Clin/uni only	Group only or both	P; or P/C; or P/C/O
Boutelle (2017)[52,53]	240	Yes	26	20	150	-0.597	Yes	Other	In-per only	Clin/uni only	Group only or both	P; or P/C; or P/C/O
Branscum (2013)[54,55]	29	Yes	4	4	30	-0.034	No	2-11 only	In-per only	Sch/com only	Group only or both	Child only
Brennan (2008)[56-59]	30	Yes	39	14	60	-0.215	Yes	Other	In-per & O	All combos	individual only	P; or P/C; or P/C/O
Bryant (2011)[60]	31	No	17.3	35		0.065	No	Other	In-per only	Sch/com only	Group only or both	P; or P/C; or P/C/O
Cao (2015)[61]	249	No	90.3			0.034	Yes	2-11 only	In-per only	Sch/com only	Group only or both	P; or P/C; or P/C/O
Chen (2010)[62]	33	No	8	8	45		Yes	2-11 only	In-per & O	Sch/com only	Group only or both	P; or P/C; or P/C/O
Chen (2011)[63]	34	No					Yes	12-18 only		All combos	individual only	P; or P/C; or P/C/O
Christie (2015)[64-66]	251	No	12	12	45		Yes	12-18 only	In-per only	Clin/uni only	Group only or both	Child only
Christison (2016)[67,68]	242	Yes	10	10	120	-0.073	Yes	Other	In-per & O	Sch/com only	Group only or both	Child only
Cohen (2016)[69,70]	252	No	34.4	6	90		Yes	2-11 only	In-per only	Clin/uni only	individual only	P; or P/C; or P/C/O
Coppins (2011)[71]	35	Yes	2	2	240	-0.378	Yes	Other	In-per only	Sch/com only	Group only or both	P; or P/C; or P/C/O
Crabtree (2010)[72]	36	No	12	4		-0.126	No	Other	In-per & O	All combos	individual	P; or P/C;

**Table S1.** Dose of behavioral intervention and intervention characteristics by study.												
	**Study No.**	**Included in Regression**	**Intervention Duration (weeks)**	**No. of Sessions**	**Length of Sessions (min)**	**Standardized Effect Size (g)**	**Intention to Treat**	**Age**	**Mode**	**Setting**	**Format**	**Participants**
											only	or P/C/O
Croker (2012)[73]	37	Yes	26	15	90	-0.208	Yes	Other	In-per only	Clin/uni only	Group only or both	P; or P/C; or P/C/O
Davis (2007)[74]	38	Yes	12	12	90	-0.185	No	12-18 only	In-per & O	All combos	individual only	P; or P/C; or P/C/O
Davis (2009)[75,76]	39	No	34.6	8	90		Yes	12-18 only	In-per & O	All combos	Group only or both	P; or P/C; or P/C/O
Davis (2011)[77]	40	No					No	2-11 only	In-per & O	Sch/com only	Group only or both	P; or P/C; or P/C/O
Davis (2013)[78]	41	No					No	2-11 only		All combos	Group only or both	P; or P/C; or P/C/O
Davis (2016)[79]	253	Yes	12	12	120	0.000	Yes	Other	In-per only	Sch/com only	Group only or both	P; or P/C; or P/C/O
Davis (2016)[80]	254	No					Yes	Other	In-per & O	Sch/com only	Group only or both	P; or P/C; or P/C/O
Davoli (2013)[81]	42	Yes	52	5	45	-0.253	Yes	2-11 only	In-per only	Clin/uni only	individual only	P; or P/C; or P/C/O
De Bock(2012)[82]	45	No	26	15	120		Yes	2-11 only	In-per only	Sch/com only	Group only or both	P; or P/C; or P/C/O
de Niet(2012)[83,84]	46	Yes	52	3		-0.441	No	Other	In-per & O	All combos	Group only or both	P; or P/C; or P/C/O
DeBar (2012)[85]	47	Yes	21.6	28	90	-0.294	Yes	12-18 only	In-per only	Clin/uni only	Group only or both	P; or P/C; or P/C/O
Delisle-Nystrom (2017)[86,87]	255	No					Yes	2-11 only		All combos	individual only	Child only
Diaz (2010)[88]	49	Yes	52	29	75.39	-0.545	Yes	Other	In-per only	Clin/uni only	Group only or both	P; or P/C; or P/C/O
Doyle (2008)[89]	50	No					Yes	12-18 only		All combos	Group only or both	P; or P/C; or P/C/O
Doyle-Baker (2011)[90]	51	Yes	10	20	60	0.022	No	2-11 only	In-per only		Group only or both	P; or P/C; or P/C/O
DreyerGillette (2014)[91,92]	256	Yes	24	48	30	-0.359	Yes	Other	In-per only	Clin/uni only	Group only or both	P; or P/C; or P/C/O
Duffy (1993)[93]	52	Yes	8	8	90	-0.058	No	Other	In-per & O		individual only	P; or P/C; or P/C/O
Duggins (2010)[94]	53	No	52	4			No	Other	In-per & O	All combos	Group only or both	P; or P/C; or P/C/O
Ebbeling (2006)[95]	54	No	25	1			Yes	12-18 only	In-per & O	All combos	individual only	P; or P/C; or P/C/O
Ebbeling (2012)[96]	55	Yes	52	3	20	0.102	Yes	12-18 only	In-per & O	All combos	individual only	P; or P/C; or P/C/O
Elder (2013)[97]	56	Yes	104	5	84	-0.038	Yes	2-11 only	In-per & O	All combos	Group only or both	P; or P/C; or P/C/O
Epstein (1994)[98]	59	No	52	32		-0.998	No	Other	In-per & O	Clin/uni only	Group only	P; or P/C;

Table S1. Dose of behavioral intervention and intervention characteristics by study.

	Study No.	Included in Regression	Intervention Duration (weeks)	No. of Sessions	Length of Sessions (min)	Standardized Effect Size (g)	Intention to Treat	Age	Mode	Setting	Format	Participants
											or both	or P/C/O
Epstein (1995)[99]	60	No	52	18			No	Other	In-per & O		Group only or both	P; or P/C; or P/C/O
Epstein (2000) a[100,101]	61	Yes	26.6	20	45	-0.510	Yes	Other	In-per & O		Group only or both	P; or P/C; or P/C/O
Epstein (2000) b[101,102]	224	Yes	26	18	22.5	-1.376	No	Other	In-per & O		Group only or both	P; or P/C; or P/C/O
Epstein (2001) a[103]	63	No	26	14	60		No	2-11 only	In-per & O	Clin/uni only	Group only or both	P; or P/C; or P/C/O
Epstein (2001) b[104]	64	No	26	20	30		No	Other	In-per & O	Clin/uni only	Group only or both	P; or P/C; or P/C/O
Epstein (2004)[105]	65	Yes	26	20	52.5	-0.910	No	Other	In-per & O		Group only or both	P; or P/C; or P/C/O
Epstein (2005)[106]	66	No	26	14		-0.562	Yes	Other	In-per only			P; or P/C; or P/C/O
Epstein (2008) a[107]	67	No					Yes	2-11 only	In-per & O	All combos	individual only	P; or P/C; or P/C/O
Epstein (2008) b[108]	225	Yes	26	13	90	-0.802	No	Other	In-per & O		Group only or both	P; or P/C; or P/C/O
Epstein (2014)[109]	68	Yes	26	15	60	-0.302	Yes	Other	In-per & O	Clin/uni only	Group only or both	P; or P/C; or P/C/O
Epstein (2015) [110]	246	No	26	15	80		Yes	Other	In-per only	Sch/com only	Group only or both	P; or P/C; or P/C/O
Esfarjani (2013)[111]	69	Yes	26	12	240	0.366	No	2-11 only	In-per only		Group only or both	P; or P/C; or P/C/O
Estabrooks (2009)[112]	70	Yes	2	2	120	-1.730	Yes	Other	In-per & O	All combos	Group only or both	P; or P/C; or P/C/O
Falbe (2015)[113]	243	Yes	10	5	120	-1.076	Yes	Other	In-per & O	Clin/uni only	Group only or both	P; or P/C; or P/C/O
Fitzgibbon (2002)[114-117]	72	Yes	14	42	45	0.066	No	2-11 only	In-per & O	Sch/com only	Group only or both	P; or P/C; or P/C/O
Fitzgibbon (2013)[118]	73	Yes	14	42	40	0.000	No	2-11 only	In-per & O	Sch/com only	Group only or both	P; or P/C; or P/C/O
Flodmark (1993)[119]	74	No	68.8	12			Yes	2-11 only	In-per only	Clin/uni only	individual only	P; or P/C; or P/C/O
Foley (2014)[120-122]	257	No					Yes	Other		All combos	individual only	Child only
Ford (2010)[18,123]	8	No	52	22		-0.527	Yes	Other	In-per & O	All combos	individual only	Child only
French (2011)[124,125]	75	No	26	6	120		No	Other	In-per & O	All combos	Group only or both	P; or P/C; or P/C/O
French (2015)[126]	244	No	26	1			No	Other	In-per & O	All combos	individual only	Child only
Frenn (2013)[127]	76	No					No	Other		All combos	individual	P; or P/C;

Table S1. Dose of behavioral intervention and intervention characteristics by study.

	Study No.	Included in Regression	Intervention Duration (weeks)	No. of Sessions	Length of Sessions (min)	Standardized Effect Size (g)	Intention to Treat	Age	Mode	Setting	Format	Participants
											only	or P/C/O
Fulkerson (2010)[128]	77	No	13	5	90		Yes	2-11 only	In-per & O	Sch/com only	Group only or both	P; or P/C; or P/C/O
Fulkerson (2015)[129-131]	258	No	43	10	120		Yes	Other	In-per & O	Sch/com only	Group only or both	P; or P/C; or P/C/O
Garipagaoglu (2009)[132]	79	Yes	13	7	30	-0.198	No	Other	In-per only	Clin/uni only	Group only or both	P; or P/C; or P/C/O
Gerards (2012)[133,134]	80	Yes	14	10	90	-0.071	Yes	2-11 only	In-per & O	All combos	Group only or both	P; or P/C; or P/C/O
Ghatrehsamani (2010)[135]	81	No	13	10		-0.072	No	Other	In-per only	Clin/uni only	Group only or both	Child only
Gillis (2007)[136]	82	Yes	13	2	30	-0.080	No	Other	In-per & O	All combos	individual only	P; or P/C; or P/C/O
Golan (1998)[137-139]	83	No	52	14	60	-6.301	No	2-11 only	In-per only	Clin/uni only	Group only or both	P; or P/C; or P/C/O
Golan (2006)[140]	85	No	26	16	60		Yes	2-11 only	In-per only	Clin/uni only	Group only or both	P; or P/C; or P/C/O
Goldfield (2001)[141]	86	Yes	20.3	13	57.5	-0.457	No	Other	In-per & O		Group only or both	P; or P/C; or P/C/O
Golley (2007)[142,143]	91	Yes	4	4	120	-0.378	Yes	2-11 only	In-per & O	All combos	Group only or both	P; or P/C; or P/C/O
Gourlan (2013)[144]	94	No	13	2	30		Yes	Other	In-per & O	All combos	individual only	Child only
Gunnarsdottir (2011)[145]	95	Yes	17.3	11	90	-0.483	No	Other	In-per & O	Clin/uni only	Group only or both	P; or P/C; or P/C/O
Guo (2015)[146]	259	Yes	52	12	120	-0.207	No	2-11 only	In-per & O	Sch/com only	Group only or both	Child only
Habib-Mourad (2014)[147,148]	260	No	26	12	45		No	2-11 only	In-per only	Sch/com only	Group only or both	Child only
Haines (2016)[149]	261	Yes	9	9	120	0.138	Yes	2-11 only	In-per only	Sch/com only	Group only or both	P; or P/C; or P/C/O
Haire-Joshu (2010)[150]	96	No	17.3	8			No	Other	In-per & O	Sch/com only	individual only	P; or P/C; or P/C/O
Harder-Lauridsen (2014)[151]	262	Yes	24	24	60	-0.515	Yes	2-11 only	In-per only	All combos	Group only or both	P; or P/C; or P/C/O
Hendrie (2011)[152,153]	97	Yes	12	3	30	-0.124	No	Other	In-per & O		individual only	P; or P/C; or P/C/O
Herget (2016)[154,155]	263	No	26	40	60		Yes	12-18 only	In-per & O	All combos	Group only or both	Child only
Hidayanty (2016)[156]	264	Yes	12	12	75	-0.215	Yes	Other	In-per & O	Sch/com only	Group only or both	P; or P/C; or P/C/O
Ho (2016)[157]	265	Yes	26	1	75	-0.350	Yes	Other	In-per & O	Clin/uni only	Group only or both	Child only
Hofsteenge (2008)[158-160]	98	Yes	13	7	90	-0.239	Yes	Other	In-per & O	Clin/uni only	Group only	P; or P/C;

Table S1. Dose of behavioral intervention and intervention characteristics by study.

	Study No.	Included in Regression	Intervention Duration (weeks)	No. of Sessions	Length of Sessions (min)	Standardized Effect Size (g)	Intention to Treat	Age	Mode	Setting	Format	Participants
											or both	or P/C/O
Hull (2016)[161,162]	266	No	17.2	8	90		Yes	2-11 only	In-per & O	All combos	Group only or both	P; or P/C; or P/C/O
Hystad (2013)[163]	100	Yes	104	15	120	-0.316	No	Other	In-per & O	Clin/uni only	Group only or both	P; or P/C; or P/C/O
Israel (1994)[164]	101	No	26	34	90		Yes	Other	In-per & O		Group only or both	P; or P/C; or P/C/O
Janicke (2008)[165-167]	102	Yes	16	24	90	-0.132	No	Other	In-per only	Sch/com only	Group only or both	P; or P/C; or P/C/O
Jansen (2011)[168]	104	Yes	10	8	120	-0.248	No	Other	In-per only	All combos	Group only or both	P; or P/C; or P/C/O
Jelalian (2006)[169-171]	105	Yes	16	32	90	-0.476	Yes	12-18 only	In-per only	All combos	Group only or both	P; or P/C; or P/C/O
Jelalian (2010)[172-175]	106	Yes	26	34	60	-0.450	Yes	12-18 only	In-per only	Clin/uni only	Group only or both	P; or P/C; or P/C/O
Jensen (2013)[176]	107	No	10	7			No	Other	In-per & O	All combos	individual only	P; or P/C; or P/C/O
Jiang (2005)[177]	108	No	104	24		-0.056	No	12-18 only	In-per & O	All combos	individual only	P; or P/C; or P/C/O
Johnson (1997)[178]	109	Yes	16	16	90	0.109	No	Other	In-per only		Group only or both	P; or P/C; or P/C/O
Jones (2007)[179-186]	110	Yes	10	20	120	-0.381	Yes	2-11 only	In-per & O	All combos	Group only or both	P; or P/C; or P/C/O
Jones (2008)[187]	223	No					Yes	12-18 only		All combos	individual only	P; or P/C; or P/C/O
Jones (2015)[188]	267	Yes	30.1	52	120	-0.056	Yes	2-11 only	In-per & O	All combos	Group only or both	Child only
Kalarchian (2009)[189,190]	111	Yes	26	20	60	0.083	Yes	Other	In-per & O	All combos	Group only or both	P; or P/C; or P/C/O
Kalavainen (2007)[191-194]	112	Yes	26	29	90	-0.489	No	2-11 only	In-per & O	Clin/uni only	Group only or both	P; or P/C; or P/C/O
Kelishadi (2008) a[195]	113	No	26	6		-3.079	No	2-11 only	In-per only	Clin/uni only	individual only	P; or P/C; or P/C/O
Kelishadi (2008) b[196]	226	No	6	18	75		No	12-18 only	In-per only	Clin/uni only	Group only or both	Child only
Khadilkar (2012)[197]	114	No	17.3	4		-2.905	Yes	Other	In-per & O	All combos	individual only	P; or P/C; or P/C/O
Khanal (2015)[198]	268	Yes	10	20	120	-0.355	Yes	Other	In-per only	All combos	Group only or both	P; or P/C; or P/C/O
Kim (2016)[199]	269	No	5	11	60		No	Other	In-per & O	All combos	Group only or both	P; or P/C; or P/C/O
Kitzman-Ulrich (2009)[200]	115	Yes	16	11		0.000	No	12-18 only	In-per only		Group only or both	P; or P/C; or P/C/O
Klesges (2008)[201-203]	116	Yes	104	34	90	0.972	Yes	2-11 only	In-per & O	All combos	Group only	P; or P/C;

Table S1. Dose of behavioral intervention and intervention characteristics by study.

	Study No.	Included in Regression	Intervention Duration (weeks)	No. of Sessions	Length of Sessions (min)	Standardized Effect Size (g)	Intention to Treat	Age	Mode	Setting	Format	Participants
											or both	or P/C/O
Kobel (2017)[204-206]	270	No	39	20		0.012	No	2-11 only	In-per only	Sch/com only	Group only or both	Child only
Kulendran (2016)[207]	271	No					No	Other		All combos	individual only	Child only
Kulik (2015)[208,209]	272	Yes	16	8	90	-0.360	Yes	12-18 only	In-per & O	Clin/uni only	Group only or both	Child only
Larsen (2015)[210]	273	Yes	104	18	30	-0.293	Yes	2-11 only	In-per only	Clin/uni only	Group only or both	Child only
Leach (2008)[211]	118	No	8.6				No	2-11 only	In-per only	All combos	Group only or both	Child only
Leme (2016)[212,213]	274	Yes	10	40	45	0.000	Yes	12-18 only	In-per & O	Sch/com only	Group only or both	Child only
Lochrie (2013)[214]	121	Yes	26	14	75	-0.635	Yes	2-11 only	In-per only	Sch/com only	Group only or both	P; or P/C; or P/C/O
Looney (2014)[215]	275	Yes	26	3	30	-0.226	Yes	2-11 only	In-per & O	All combos	individual only	Child only
Love-Osborne (2014)[216]	276	No	34.4	8			No	12-18 only	In-per & O	All combos	individual only	Child only
Lubans (2016)[217-219]	277	Yes	34.4	9	20	0.008	Yes	12-18 only	In-per & O	Sch/com only	Group only or both	Child only
Luszczynska (2016)[220]	278	No	8.3	9	60		No	12-18 only	In-per only	Sch/com only	Group only or both	Child only
Macdonell (2012)[221]	122	No	10	4	60		No	12-18 only	In-per only	Clin/uni only	individual only	P; or P/C; or P/C/O
Macias-Cervantes (2009)[222]	123	Yes	12	36	60	0.143	No	2-11 only	In-per only	Sch/com only	Group only or both	Child only
Maddison (2011)[223,224]	125	Yes	1	1	60	0.012	Yes	Other	In-per & O	All combos	individual only	P; or P/C; or P/C/O
Madsen (2013)[225]	126	No	12	60	60		No	2-11 only	In-per only	Sch/com only	Group only or both	Child only
Madsen (2015)[226,227]	279	No	12	12		-0.047	No	2-11 only	In-per only	Sch/com only	Group only or both	Child only
Magarey (2011)[228]	127	Yes	26	12	105	-0.415	Yes	2-11 only	In-per & O	All combos	Group only or both	P; or P/C; or P/C/O
Majumdar (2015)[229]	280	Yes	6	1	60	0.193	Yes	Other	In-per & O	Clin/uni only	individual only	P; or P/C; or P/C/O
Markert (2013)[230,231]	128	No					Yes	Other		All combos	individual only	P; or P/C; or P/C/O
Martinez-Vizcaino (2008)[232,233]	129	No	104	149	90		Yes	2-11 only	In-per only	Sch/com only	Group only or both	Child only
Martinez-Andrade (2014)[234]	130	Yes	6	6	120	-0.182	Yes	2-11 only	In-per & O	Clin/uni only	Group only or both	P; or P/C; or P/C/O
Martinez-Vizcaino (2014)[235,236]	131	No	39	80	110.25		Yes	2-11 only	In-per & O	Sch/com	Group only	P; or P/C;

Table S1. Dose of behavioral intervention and intervention characteristics by study.

	Study No.	Included in Regression	Intervention Duration (weeks)	No. of Sessions	Length of Sessions (min)	Standardized Effect Size (g)	Intention to Treat	Age	Mode	Setting	Format	Participants
										only	or both	or P/C/O
McCallum(2005)[237-239]	132	Yes	12	4	21.87	-0.065	Yes	2-11 only	In-per & O	Clin/uni only	individual only	P; or P/C; or P/C/O
Melnyk (2015)[240,241]	305	No	15	15	75		Yes	12-18 only	In-per & O	Sch/com only	Group only or both	Child only
Mendoza (2016)[242]	281	No	8	7	22.5		No	2-11 only	In-per & O	Sch/com only	Group only or both	Child only
Moens (2012)[243]	134	No	21.6	6	120		Yes	Other	In-per & O		Group only or both	P; or P/C; or P/C/O
Morgan (2011)[244-246]	135	Yes	13	8	75	0.000	Yes	Other	In-per & O	Clin/uni only	Group only or both	P; or P/C; or P/C/O
Munsch (2008)[247]	136	No	36	16	120		Yes	Other	In-per only	Clin/uni only	Group only or both	P; or P/C; or P/C/O
Naar-King (2009)[248,249]	137	No	26	65			No	12-18 only	In-per only	All combos	individual only	P; or P/C; or P/C/O
Nemet (2006)[250]	141	Yes	13	6	40	-0.947	No	Other	In-per & O	Clin/uni only	Group only or both	P; or P/C; or P/C/O
Nemet (2008)[251]	142	Yes	13	53	56.6	-0.295	Yes	2-11 only	In-per & O	Clin/uni only	Group only or both	P; or P/C; or P/C/O
Nemet (2013) a[252]	143	Yes	12	6	40	-0.445	No	Other	In-per & O	Clin/uni only	Group only or both	P; or P/C; or P/C/O
Nemet (2013) b[253]	227	Yes	12	6	62.5	-0.564	No	2-11 only	In-per & O	Clin/uni only	Group only or both	P; or P/C; or P/C/O
Neumark-Sztainer (2009)[254]	144	No	7	14	120		Yes	Other	In-per & O	Sch/com only	Group only or both	P; or P/C; or P/C/O
Nollen (2014)[255]	145	No					Yes	Other		All combos	individual only	Child only
Nourian (2017)[256]	286	Yes	13	12	60	-0.115	Yes	12-18 only	In-per & O	Clin/uni only	Group only or both	Child only
Nova (2001)[257]	146	No	104	9			Yes	Other	In-per & O	Clin/uni only	individual only	P; or P/C; or P/C/O
Novotny (2015)[258]	287	Yes	65	5	150	0.162	No	2-11 only	In-per & O	Clin/uni only	individual only	P; or P/C; or P/C/O
Nyberg (2016)[259]	288	No	26	10	30		Yes	2-11 only	In-per & O	Sch/com only	Group only or both	P; or P/C; or P/C/O
O'Conner (2013)[260,261]	147	No	26	6			No	2-11 only	In-per & O	All combos	individual only	P; or P/C; or P/C/O
Ostbye (2011)[262,263]	148	No	1	1		0.030	Yes	2-11 only	In-per & O	All combos	Group only or both	P; or P/C; or P/C/O
Paineau (2008)[264]	149	No					Yes	2-11 only		All combos	individual only	P; or P/C; or P/C/O
Pakpour (2015)[265]	291	Yes	6	6	40	-0.388	Yes	12-18 only	In-per only	Clin/uni only	Group only or both	P; or P/C; or P/C/O
Papadaki (2010)[266,267]	150	No	26	6		-0.053	Yes	Other	In-per only	All combos	individual	P; or P/C;

Table S1. Dose of behavioral intervention and intervention characteristics by study.

	Study No.	Included in Regression	Intervention Duration (weeks)	No. of Sessions	Length of Sessions (min)	Standardized Effect Size (g)	Intention to Treat	Age	Mode	Setting	Format	Participants
											only	or P/C/O
Parra-Medina (2015)[268]	292	No	18	5	42		Yes	Other	In-per & O	All combos	individual only	P; or P/C; or P/C/O
Patrick (2006)[269]	151	No	1	1			Yes	Other	In-per & O	All combos	individual only	P; or P/C; or P/C/O
Patrick (2013)[270]	152	No					Yes	12-18 only		All combos	individual only	P; or P/C; or P/C/O
Pbert (2016)[271]	293	Yes	34	12	30	-0.088	Yes	12-18 only	In-per only	Sch/com only	Group only or both	Child only
Pedrosa (2011)[272,273]	153	No	52	4		-0.560	Yes	2-11 only	In-per only	Clin/uni only	individual only	P; or P/C; or P/C/O
Quattrin (2012)[274,275]	155	Yes	104	16	60	-1.288	Yes	2-11 only	In-per & O	All combos	Group only or both	P; or P/C; or P/C/O
Raynor (2002)[276]	156	No	26	13	60		No	Other	In-per only	Clin/uni only	Group only or both	P; or P/C; or P/C/O
Raynor (2012)[277] – Trial 1	157	Yes	26	8	45	-0.225	Yes	2-11 only	In-per & O	Clin/uni only	Group only or both	P; or P/C; or P/C/O
Raynor (2012)[277] – Trial 2	228	No	26	8	45		Yes	2-11 only	In-per & O	Clin/uni only	Group only or both	P; or P/C; or P/C/O
Reinehr (2010)[278-280]	158	Yes	26	48	83	-0.853	Yes	Other	In-per only	Clin/uni only	Group only or both	P; or P/C; or P/C/O
Rerksuppaphol (2017)[281]	294	No					Yes	2-11 only	In-per & O	Sch/com only	individual only	Child only
Resnick (2009)[282]	159	No	18			-0.299	Yes	2-11 only	In-per & O	All combos	individual only	P; or P/C; or P/C/O
Resnicow (2005)[283]	160	No	26	23		0.042	Yes	12-18 only	In-per & O	All combos	Group only or both	P; or P/C; or P/C/O
Resnicow (2012)[284,285]	161	No	104	10			Yes	2-11 only	In-per & O	All combos	individual only	P; or P/C; or P/C/O
Robertson (2017)[286,287]	308	Yes	10	10	150	-0.028	Yes	2-11 only	In-per only	Sch/com only	Group only or both	P; or P/C; or P/C/O
Robinson (2003)[288]	162	No	12	58	150		Yes	2-11 only	In-per & O	All combos	Group only or both	P; or P/C; or P/C/O
Robinson (2008)[289,290]	163	No	104	500	150	0.241	Yes	2-11 only	In-per only	All combos	Group only or both	P; or P/C; or P/C/O
Rodearmel (2006)[291]	164	No	13	3		-0.090	No	Other	In-per & O	All combos	individual only	P; or P/C; or P/C/O
Rodearmel (2007)[292,293]	165	No					Yes	Other			individual only	P; or P/C; or P/C/O
Roemmich (2004)[294]	166	No	6	6			Yes	Other	In-per only	All combos	individual only	P; or P/C; or P/C/O
Rooney (2005)[295]	167	No	12	6	60		Yes	Other	In-per & O		individual only	P; or P/C; or P/C/O
Rosado (2008)[296]	168	No	12	12		-0.073	Yes	Other	In-per & O	Sch/com	individual	P; or P/C;

**Table S1.** Dose of behavioral intervention and intervention characteristics by study.												
	Study No.	Included in Regression	Intervention Duration (weeks)	No. of Sessions	Length of Sessions (min)	Standardized Effect Size (g)	Intention to Treat	Age	Mode	Setting	Format	Participants
										only	only	or P/C/O
Rosenkranz (2010)[297]	169	Yes	17.3	8	75	-0.021	No	Other	In-per only	Sch/com only	Group only or both	Child only
Ruotsalainen (2015)[298]	295	No					Yes	12-18 only		All combos	Group only or both	P; or P/C; or P/C/O
Sacher (2010)[299]	170	Yes	9	18	120	-0.581	Yes	Other	In-per only	Sch/com only	Group only or both	P; or P/C; or P/C/O
Saelens (2002)[300]	171	No	1	1		0.041	Yes	12-18 only	In-per & O	All combos	individual only	P; or P/C; or P/C/O
Saelens (2011)[301]	172	Yes	14	42	37.3	-0.385	Yes	2-11 only	In-per only	Clin/uni only	Group only or both	P; or P/C; or P/C/O
Saelens (2013)[302]	173	Yes	22	60	30	-1.271	Yes	2-11 only	In-per only	Clin/uni only	Group only or both	P; or P/C; or P/C/O
Savoye (2007)[303-305]	174	Yes	26	26	40	-0.466	Yes	Other	In-per only	Sch/com only	Group only or both	P; or P/C; or P/C/O
Savoye (2014)[306]	229	Yes	26	53	50	-0.122	Yes	Other	In-per only		Group only or both	P; or P/C; or P/C/O
Serra-Paya (2013)[307,308]	175	Yes	30	90	60	-0.232	Yes	Other	In-per only	All combos	Group only or both	P; or P/C; or P/C/O
Sharifah (2011)[309,310]	176	Yes	26	16	60	0.000	No	2-11 only	In-per only	Clin/uni only	Group only or both	P; or P/C; or P/C/O
Shelton (2007)[311]	177	No	4.3	4	120		No	2-11 only	In-per & O	Sch/com only	Group only or both	P; or P/C; or P/C/O
Sherwood (2015)[312]	296	Yes	1	1	10	-0.053	No	2-11 only	In-per & O	All combos	individual only	P; or P/C; or P/C/O
Shrewsbury (2009)[313-318]	178	No					Yes	12-18 only		All combos	Group only or both	P; or P/C; or P/C/O
Siwik (2013)[319]	180	No	12	12	90		Yes	2-11 only	In-per & O	Clin/uni only	Group only or both	P; or P/C; or P/C/O
Skouteris (2010)[320,321]	181	Yes	10	10	90	0.000	Yes	2-11 only	In-per & O	Sch/com only	Group only or both	P; or P/C; or P/C/O
Slusser (2012)[322]	182	Yes	16	9	90	-0.234	Yes	2-11 only	In-per & O	All combos	Group only or both	P; or P/C; or P/C/O
Small (2013)[323]	183	Yes	16	4	45	-0.302	Yes	2-11 only	In-per & O	All combos	individual only	P; or P/C; or P/C/O
Stark (2011)[324,325]	184	Yes	26	18	90	-0.529	Yes	2-11 only	In-per only	All combos	Group only or both	P; or P/C; or P/C/O
Steele (2012)[326,327]	185	No	10	20	90		Yes	Other	In-per only	Clin/uni only	Group only or both	P; or P/C; or P/C/O
Stettler (2014)[328]	309	Yes	52	12	20	-0.116	Yes	Other	In-per only	Clin/uni only	individual only	P; or P/C; or P/C/O
Stewart (2005)[329,330]	186	No	26	8	37.5		Yes	2-11 only	In-per only	All combos	individual only	P; or P/C; or P/C/O
Stolley (1997)[331]	187	No	12	12	60		Yes	Other	In-per only	Sch/com	Group only	P; or P/C;

**Table S1.** Dose of behavioral intervention and intervention characteristics by study.												
	**Study No.**	**Included in Regression**	**Intervention Duration (weeks)**	**No. of Sessions**	**Length of Sessions (min)**	**Standardized Effect Size (g)**	**Intention to Treat**	**Age**	**Mode**	**Setting**	**Format**	**Participants**
										only	or both	or P/C/O
Story (2003)[332]	188	No	12	24	60		No	2-11 only	In-per & O	All combos	Group only or both	P; or P/C; or P/C/O
Story (2012)[333,334]	189	Yes	91	225	60	0.112	Yes	2-11 only	In-per & O	All combos	Group only or both	P; or P/C; or P/C/O
Stovitz (2014)[335]	190	No	1	1		-0.036	Yes	2-11 only	In-per & O	All combos	individual only	P; or P/C; or P/C/O
Tanofsky-Kraff (2017)[336,337]	307	No	13	13	90	-0.459	Yes	12-18 only	In-per only	Clin/uni only	Group only or both	Child only
Tanofsky Kraff (2010)[338]	191	Yes	12	12	90	-0.202	Yes	12-18 only	In-per only	Clin/uni only	Group only or both	Child only
Taveras (2011)[339-342]	192	No	52	4	25		Yes	2-11 only	In-per & O	All combos	individual only	P; or P/C; or P/C/O
Taveras (2012)[343,344]	193	Yes	24	4	60	-0.054	Yes	2-11 only	In-per & O	All combos	individual only	P; or P/C; or P/C/O
Taveras (2013)[345,346]	194	No					Yes	Other		All combos	individual only	P; or P/C; or P/C/O
Taveras (2017)[347,348]	304	Yes	52	1	30	-0.140	Yes	Other	In-per & O	All combos	individual only	Child only
Taylor (2010)[349,350]	195	Yes	104	14	35	-0.501	Yes	2-11 only	In-per & O	All combos	individual only	P; or P/C; or P/C/O
Tjonna (2009)[351]	197	Yes	13	6	210	0.048	No	12-18 only	In-per only	Clin/uni only	Group only or both	Child only
Tomayko (2016)[352,353]	298	Yes	52	12	60	0.098	Yes	2-11 only	In-per & O	All combos	Group only or both	Child only
Toulabi (2012)[354]	198	No		30	56		Yes	12-18 only	In-per & O	Sch/com only	Group only or both	P; or P/C; or P/C/O
Trost (2014)[355]	299	Yes	16	16	60	-0.416	Yes	Other	In-per & O	All combos	Group only or both	Child only
Tsiros (2008)[356]	222	Yes	10	9	60	0.020	Yes	12-18 only	In-per & O	All combos	Group only or both	P; or P/C; or P/C/O
Veldhuis (2009)[357,358,359]	200	No	52	3	30		Yes	2-11 only	In-per & O	Clin/uni only	individual only	P; or P/C; or P/C/O
Vos (2011)[360-363]	201	Yes	52	15	150	-0.361	Yes	Other	In-per & O	Clin/uni only	Group only or both	P; or P/C; or P/C/O
Wadden (1990)[364]	202	Yes	16	32	60	0.071	No	12-18 only	In-per & O	Clin/uni only	Group only or both	P; or P/C; or P/C/O
Wake (2009)[365]	203	No	12	4			No	2-11 only	In-per & O	Clin/uni only	individual only	P; or P/C; or P/C/O
Wake (2012)[366,367]	204	Yes	65	13	17.9	-0.435	Yes	2-11 only	In-per only	Clin/uni only	individual only	P; or P/C; or P/C/O
Waling (2010)[368-372]	205	Yes	52	14	105	0.166	Yes	Other	In-per & O	Clin/uni only	Group only or both	P; or P/C; or P/C/O
Walpole (2013)[373]	206	Yes	26	6	30	-0.102	No	Other	In-per only	Clin/uni only	individual	P; or P/C;

**Table S1.** Dose of behavioral intervention and intervention characteristics by study.												
	**Study No.**	**Included in Regression**	**Intervention Duration (weeks)**	**No. of Sessions**	**Length of Sessions (min)**	**Standardized Effect Size (g)**	**Intention to Treat**	**Age**	**Mode**	**Setting**	**Format**	**Participants**
											only	or P/C/O
Walton (2016)[374]	300	No	9	9	120		Yes	2-11 only	In-per only	Sch/com only	Group only or both	P; or P/C; or P/C/O
Warren (2003)[375]	207	No	60.2	20	25		Yes	2-11 only	In-per & O	Sch/com only	Group only or both	P; or P/C; or P/C/O
Weigel (2008)[376]	209	Yes	52	24	52.5	-0.674	No	Other	In-per only	Clin/uni only	Group only or both	P; or P/C; or P/C/O
Weigensberg (2014)[377]	210	Yes	12	12	90	0.095	Yes	12-18 only	In-per only	Sch/com only	Group only or both	Child only
Weintraub (2008)[378]	211	Yes	26	86	135	-0.167	Yes	2-11 only	In-per only	Sch/com only	Group only or both	Child only
Wengle (2011)[379]	212	Yes	24	24	90	-0.067	No	12-18 only	In-per & O	All combos	individual only	P; or P/C; or P/C/O
West (2010)[380]	213	Yes	12	9	90	-0.248	Yes	2-11 only	In-per & O		Group only or both	P; or P/C; or P/C/O
White (2004)[381-383]	214	No	12	4		0.073	Yes	Other	In-per & O	All combos	individual only	P; or P/C; or P/C/O
Wilfley (2007)[384-387]	215	Yes	16	32	40	-0.084	Yes	Other	In-per only	Clin/uni only	Group only or both	P; or P/C; or P/C/O
Willeboordse (2016)[388,389]	301	Yes	80	18	82.5	-0.236	Yes	Other	In-per only	Clin/uni only	Group only or both	Child only
Wright (2012)[390-392]	217	No	6	6	90		Yes	Other	In-per & O	Sch/com only	Group only or both	P; or P/C; or P/C/O
Wright (2013)[393]	218	No	1	1		-0.195	Yes	Other	In-per & O	All combos	individual only	P; or P/C; or P/C/O
Yackobovitch-Gavan (2009)[394,395]	219	Yes	12	36	90	-0.670	No	2-11 only	In-per only	Clin/uni only	Group only or both	P; or P/C; or P/C/O
Yilmaz (2015)[396]	302	No					No	2-11 only		All combos	individual only	P; or P/C; or P/C/O
Yin (2005) a[397]	220	Yes	1	1	150	0.000	No	2-11 only	In-per & O	Sch/com only	Group only or both	Child only
Yin (2005) b[398-403]	221	No	34.6	128	80		Yes	2-11 only	In-per only	Sch/com only	Group only or both	Child only

Study number was assigned by the study team and can be used to reference the reporting of effect size in the forest plot.
Mode: Studies were coded as 1) in-person only (In-per only) or 2) in-person and other mode (In-per & O)
Setting: Studies were coded as 1) Clinic/University Only (Clin/uni only), 2) School/Community only (sch/com only), or 3) All other combinations (All combos)
Format: Studies were coded as 1)Individual only, or 2)Group only or both
Participants: Studies were coded as 1) Child only or 2) Parent only, Parent/child or parent/child/other.
Blank fields represent data that could not be extracted from the articles referenced.

Table S2: Meta-regression evaluating the relationship between dose and standardized effect size stratified by study characteristics of intent to treat (yes vs. no), mode (in person only vs. in person + other mode), setting (clinic or university vs. school or community, vs. combination), format (individual only vs. group or individual + group), and baseline weight status (normal weight & overweight/obese vs. overweight/obese only).

**Intent to treat:**	Coefficient	95% CI	p-value
No (n = 44)			
Contact Hours	-0.002	-0.011, 0.007	0.7
Duration	-0.002	-0.008, 0.004	0.5
Contact Hours x Duration	0.000	0.000, 0.001	0.6
Yes (n = 89)			
Contact Hours	0.000	-0.003, 0.002	0.2
Duration	-0.003	-0.007, 0.002	0.2
Contact Hours x Duration	0.000	0.000, 0.000	0.3
**Mode:**			
In Person Only (n = 57)			
Contact Hours	-0.001	-0.003, 0.001	0.4
Duration	-0.003	-0.007, 0.002	0.2
Contact Hours x Duration	0.000	0.000, 0.000	0.3
In Person + Other Mode (n = 76)			
Contact Hours	0.000	-0.005, 0.005	0.9
Duration	0.001	-0.002, 0.005	0.5
Contact Hours x Duration	0.000	0.000, 0.000	0.4
**Setting:**			
Clinic or University (n = 55)			
Contact Hours	-0.004	-0.008, 0.000	0.06
Duration	0.001	-0.003, 0.004	0.7
Contact Hours x Duration	0.000	0.000, 0.000	0.2

School or Community (n = 27)			
Contact Hours	0.000	-0.003, 0.003	0.9
Duration	-0.004	-0.014, 0.005	0.4
Contact Hours x Duration	0.000	0.000, 0.000	0.8
Combination (n = 38)			
Contact Hours	-0.001	-0.006, 0.005	0.8
Duration	0.002	-0.003, 0.007	0.4
Contact Hours x Duration	0.000	0.000, 0.000	0.4
**Format:**			
Individual Only (n = 26)			
Contact Hours	0.000	-0.009, 0.010	0.9
Duration	0.003	-0.011, 0.018	0.7
Contact Hours x Duration	0.000	0.000, 0.001	0.4
Group or Individual + Group (n = 106)			
Contact Hours	0.000	-0.003, 0.003	0.9
Duration	0.000	-0.003, 0.004	0.8
Contact Hours x Duration	0.000	0.000, 0.000	0.3
**Baseline Weight Status:**			
Normal Weight & Overweight/Obese (n=28)			
Contact Hours	-0.001	-0.005 0.004	0.8
Duration	0.004	0.001 0.007	0.02
Contact Hours x Duration	0.000	0.000 0.000	0.8
Overweight/Obese Only (n=105)			
Contact Hours	0.000	-0.003 0.002	0.9
Duration	-0.002	-0.005 0.002	0.3
Contact Hours x Duration	0.000	0.000 0.000	0.5

REFERENCES

1. Akdemir M, Donmez L, Polat H. The effect of nutritional and physical activity interventions on nutritional status and obesity in primary school children: A cluster randomized controlled study. *Kuwait Medical Journal.* 2017;49(2):105-113.
2. Andrade S, Lachat C, Ochoa-Aviles A, et al. A school-based intervention improves physical fitness in Ecuadorian adolescents: a cluster-randomized controlled trial. *International Journal of Behavioral Nutrition and Physical Activity.* 2014;11.
3. Andrade S, Verloigne M, Cardon G, et al. School-based intervention on healthy behaviour among Ecuadorian adolescents: effect of a cluster-randomized controlled trial on screen-time. *BMC Public Health.* 2015;15.
4. Andrade S, Lachat C, Cardon G, et al. Two years of school-based intervention program could improve the physical fitness among Ecuadorian adolescents at health risk: subgroups analysis from a cluster-randomized trial. *BMC Pediatr.* 2016;16.
5. Andre N, Beguier S. Using motivational interviewing as a supplement to physical activity program in obese adolescents: a RCT study. *Eat Weight Disord-St.* 2015;20(4):519-523.
6. Arauz Boudreau AD, Kurowski DS, Gonzalez WI, Dimond MA, Oreskovic NM. Latino families, primary care, and childhood obesity: a randomized controlled trial. *Am J Prev Med.* 2013;44:S247-257.
7. Crespo NC, Elder JP, Ayala GX, et al. Results of a multi-level intervention to prevent and control childhood obesity among Latino children: the Aventuras Para Niños Study. *Ann Behav Med.* 2012;43:84-100.
8. Ayala GX, Elder JP, Campbell NR, et al. Longitudinal intervention effects on parenting of the Aventuras para Niños study. *Am J Prev Med.* 2010;38:154-162.
9. Balagopal P, George D, Sweeten S, et al. Response of fractional synthesis rate (FSR) of fibrinogen, concentration of D-dimer and fibrinolytic balance to physical activity-based intervention in obese children. *J Thromb Haemost.* 2008;6:1296-1303.
10. Balagopal P, George D, Patton N, et al. Lifestyle-only intervention attenuates the inflammatory state associated with obesity: a randomized controlled study in adolescents. *J Pediatr.* 2005;146:342-348.
11. Balagopal P, George D, Yarandi H, Funanage V, Bayne E. Reversal of obesity-related hypoadiponectinemia by lifestyle intervention: a controlled, randomized study in obese adolescents. *J Clin Endcriol Metab.* 2005;90:6192-6197.
12. Balagopal P, Bayne E, Sager B, Russell L, Patton N, George D. Effect of lifestyle changes on whole-body protein turnover in obese adolescents. *Int J Obes (Lond).* 2003;27:1250-1257.
13. Balagopal P, Gidding SS, Buckloh LM, et al. Changes in circulating satiety hormones in obese children: A randomized controlled physical activity-based intervention study. *Obesity (Silver Spring).* 2010;18:1747-1753.
14. Balagopal P, Graham TE, Kahn BB, Altomare A, Funanage V, George D. Reduction of elevated serum retinol binding protein in obese children by lifestyle intervention: association with subclinical inflammation. *J Clin Endcriol Metab.* 2007;92:1971-1974.
15. Ball GD, Mackenzie-Rife KA, Newton MS, et al. One-on-one lifestyle coaching for managing adolescent obesity: Findings from a pilot, randomized controlled trial in a real-world, clinical setting. *Paediatr Child Health.* 2011;16:345-350.
16. Bani Salameh A, Al-Sheyab N, El-Hneiti M, Shaheen A, Williams LM, Gallagher R. Effectiveness of a 12-week school-based educational preventive programme on weight and fasting blood glucose in "at-risk" adolescents of type 2 diabetes mellitus: Randomized controlled trial. *Int J Nurs Pract.* 2017;23(3).
17. Banks J, Sharp DJ, Hunt LP, Shield JP. Evaluating the transferability of a hospital-based childhood obesity clinic to primary care: a randomised controlled trial. *Br J Gen Pract.* 2012;62:e6-12.
18. Hollinghurst S, Hunt LP, Banks J, Sharp DJ, Shield JP. Cost and effectiveness of treatment options for childhood obesity. *Pediatr Obes.* 2014;9:e26-34.
19. Baranowski T, Baranowski JC, Cullen KW, et al. The Fun, Food, and Fitness Project (FFFP): the Baylor GEMS pilot study. *Ethn Dis.* 2003;13:S30-39.

20. Taylor WC, Baranowski T, Klesges LM, et al. Psychometric properties of optimism and pessimism: results from the Girls' Health Enrichment Multisite Studies. *Prev Med.* 2004;38 Suppl:S69-77.
21. Baranowski T, Baranowski J, Thompson D, et al. Video game play, child diet, and physical activity behavior change a randomized clinical trial. *Am J Prev Med.* 2011;40:33-38.
22. Barbeau P, Johnson MH, Howe CA, et a. Ten Months of exercise improves general and visceral adiposity, bone, and fitness in black girls. *Obes Res.* 2007;15:2077-2085.
23. Barkin SL, Gesell SB, Poe EK, Ip EH. Changing overweight Latino preadolescent body mass index: the effect of the parent-child dyad. *Clin Pediatr (Phila).* 2011;50:29-36.
24. Barnes AT, Plotnikoff RC, Collins CE, Morgan PJ. Feasibility and Preliminary Efficacy of the MADE4Life Program: A Pilot Randomized Controlled Trial. *J Phys Act Health.* 2015;12(10):1378-1393.
25. Bathrellou E, Yannakoulia M, Papanikolaou K, et al. Parental involvement does not augment the effectiveness of an intense behavioral program for the treatment of childhood obesity. *Hormones Athens.* 2010;9:171-175.
26. Bathrellou E, Yannakoulia M, Papanikolaou K, et al. Development of a multi-disciplinary intervention for the treatment of childhood obesity based on cognitive behavioral therapy. *Child & Family Behavior Therapy.* 2010;32:34-50.
27. Bean MK, Wilson DB, Thornton LM, Kelly N, Mazzeo SE. Dietary intake in a randomized-controlled pilot of NOURISH: a parent intervention for overweight children. *Prev Med.* 2012;55:224-227.
28. Mazzeo SE, Kelly NR, Stern M, et al. Nourishing Our Understanding of Role Modeling to Improve Support and Health (NOURISH): design and methods. *Contemp Clin Trials.* 2012;33:515-522.
29. Mazzeo SE, Kelly NR, Stern M, et al. Parent skills training to enhance weight loss in overweight children: evaluation of NOURISH. *Eat Behav.* 2014;15(2):225-229.
30. Beech BM, Klesges RC, Kumanyika SK, et al. Child- and parent-targeted interventions: the Memphis GEMS pilot study. *Ethn Dis.* 2003;13:S40-53.
31. Benestad B, Lekhal S, Smastuen MC, et al. Camp-based family treatment of childhood obesity: randomised controlled trial. *Arch Dis Child.* 2017;102(4):303-310.
32. Berkowitz RI, Rukstalis MR, BishopGilyard CT, et al. Treatment of adolescent obesity comparing self-guided and group lifestyle modification programs: A potential model for primary care. *J Pediatr Psychol.* 2013;38:978-986.
33. Berntsen S, Mowinckel P, Carlsen KH, et al. Obese children playing towards an active lifestyle. *Int J Pediatr Obes.* 2010;5:64-71.
34. Berry D, Colindres M, Sanchez-Lugo L, Sanchez M, Neal M, Smith-Miller C. Adapting, Feasibility Testing, and Pilot Testing a Weight Management Intervention for Recently Immigrated Spanish-Speaking Women and Their 2- to 4-Year-Old Children. *Hispanic Health Care Int* 2011.;9:186-193.
35. Berry DC, Schwartz TA, McMurray RG, et al. The family partners for health study: a cluster randomized controlled trial for child and parent weight management. *Nutr Diabetes.* 2014;4:e101.
36. Berry DC, McMurray R, Schwartz TA, et al. Rationale, design, methodology and sample characteristics for the family partners for health study: a cluster randomized controlled study. *BMC Public Health.* 2012;12(1):1.
37. Birken CS, Maguire J, Mekky M, et al. Office-based randomized controlled trial to reduce screen time in preschool children. *Pediatrics.* 2012;130:1110-1115.
38. Black MM, Hager ER, Le K, et al. Challenge! Health promotion/obesity prevention mentorship model among urban, black adolescents. *Pediatrics.* 2010;126:280-288.
39. Bloom T, Sharpe L, Mullan B, Zucker N. A pilot evaluation of appetite-awareness training in the treatment of childhood overweight and obesity: a preliminary investigation. *Int J Eat Disord.* 2013;46:47-51.
40. Bocca G, Corpeleijn E, van d, Heuvel, E R, Stolk RP, Sauer PJ. Three-year follow-up of 3-year-old to 5-year-old children after participation in a multidisciplinary or a usual-care obesity treatment program. *Clin Nutr.* 2013;33:1095-1100.
41. Bocca G, Corpeleijn E, Stolk RP, Sauer PJ. Results of a multidisciplinary treatment program in 3-year-old to 5-year-old overweight or obese children: a randomized controlled clinical trial. *Arch Pediatr Adolesc Med.* 2012;166:1109-1115.

42. Bocca G, Kuitert MW, Sauer PJ, Stolk RP, Flapper BC, Corpeleijn E. A multidisciplinary intervention programme has positive effects on quality of life in overweight and obese preschool children. *Acta Paediatr.* 2014;103(9):962-967.
43. Bohlin A, Hagman E, Klaesson S, Danielsson P. Childhood obesity treatment: telephone coaching is as good as usual care in maintaining weight loss - a randomized controlled trial. *Clin Obes.* 2017;7(4):199-205.
44. Danielsson P, Bohlin A, Bendito A, Svensson A, Klaesson S. Five-year outpatient programme that provided children with continuous behavioural obesity treatment enjoyed high success rate. *Acta Paediatr.* 2016;105(10):1181-1190.
45. Bonham MP, Dordevic AL, Ware RS, Brennan L, Truby H. Evaluation of a Commercially Delivered Weight Management Program for Adolescents. *J Pediatr-Us.* 2017;185:73-+.
46. Bonham MP, Dordevic AL, Ware RS, Truby H. Evaluation of 'JenMe', a Commercially-Delivered Weight Management Program for Adolescents: a Randomised Controlled Trial. *Faseb Journal.* 2017;31.
47. Dordevic AL, Bonham MP, Ware RS, Brennan L, Truby H. Study protocol: evaluation of 'JenMe', a commercially-delivered weight management program for adolescents: a randomised controlled trial. *BMC Public Health.* 2015;15.
48. Boodai SA, McColl JH, Reilly JJ. National Adolescent Treatment Trial for Obesity in Kuwait (NATTO): project design and results of a randomised controlled trial of a good practice approach to treatment of adolescent obesity in Kuwait. *Trials.* 2014;15.
49. Boutelle KN, Cafri G, Crow SJ. Parent-only treatment for childhood obesity: a randomized controlled trial. *Obesity (Silver Spring).* 2011;19:574-580.
50. Boutelle KN, Norman GJ, Rock CL, Rhee KE, Crow SJ. Guided self-help for the treatment of pediatric obesity. *Pediatrics.* 2013;131:e1435-1442.
51. Boutelle KN, Zucker N, Peterson CB, Rydell S, Carlson J, Harnack LJ. An intervention based on Schachter's externality theory for overweight children: the regulation of cues pilot. *J Pediatr Psychol.* 2014;39(4):405-417.
52. Boutelle KN, Rhee KE, Liang J, et al. Effect of Attendance of the Child on Body Weight, Energy Intake, and Physical Activity in Childhood Obesity Treatment: A Randomized Clinical Trial. *JAMA Pediatr.* 2017;171(7):622-628.
53. Boutelle KN, Braden A, Douglas JM, et al. Design of the FRESH study: A randomized controlled trial of a parent-only and parent-child family-based treatment for childhood obesity. *Contemp Clin Trials.* 2015;45:364-370.
54. Branscum P, Sharma M, Wang LL, Wilson B, Rojas-Guyler L. A process evaluation of a social cognitive theory-based childhood obesity prevention intervention: the Comics for Health program. *Health Promot Pract.* 2013;14:189-198.
55. Branscum P, Sharma M, Wang LL, Wilson BR, RojasGuyler L. A true challenge for any superhero: An evaluation of a comic book obesity prevention program. *Fam Community Health.* 2013;36:63-76.
56. Brennan L, Walkley J, Wilks R, Fraser SF, Greenway K. Physiological and behavioural outcomes of a randomised controlled trial of a cognitive behavioural lifestyle intervention for overweight and obese adolescents. *Obes Res Clin Pract.* 2013;7:e23-41.
57. Brennan L, Walkley J, Wilks R. Parent- and adolescent-reported barriers to participation in an adolescent overweight and obesity intervention. *Obesity (Silver Spring).* 2012;20:1319-1324.
58. Brennan L, Walkley J, Fraser SF, Greenway K, Wilks R. Motivational interviewing and cognitive behaviour therapy in the treatment of adolescent overweight and obesity: study design and methodology. *Contemp Clin Trials.* 2008;29:359-375.
59. Brennan L, Wilks R, Walkley J, Fraser SF, Greenway K. Treatment acceptability and psychosocial outcomes of a randomised controlled trial of a cognitive behavioural lifestyle intervention for overweight and obese adolescents. *Behaviour Change.* 2013;29:36-62.
60. Bryant M, Farrin A, Christie D, Jebb SA, Cooper AR, Rudolf M. Results of a feasibility randomised controlled trial (RCT) for WATCH IT: a programme for obese children and adolescents. *Clin Trials.* 2011;8:755-764.

61. Cao ZJ, Wang SM, Chen Y. A Randomized Trial of Multiple Interventions for Childhood Obesity in China. *Am J Prev Med.* 2015;48(5):552-560.
62. Chen JL, Weiss S, Heyman MB, Lustig RH. Efficacy of a child-centred and family-based program in promoting healthy weight and healthy behaviors in Chinese American children: a randomized controlled study. *J Public Health Oxf.* 2010;32:219-229.
63. Chen JL, Weiss S, Heyman MB, Cooper B, Lustig RH. The efficacy of the web-based childhood obesity prevention program in Chinese American adolescents (Web ABC study). *J Adolesc Health.* 2011;49:148-154.
64. Christie D, Hudson L, Costa S, et al. Effects of a motivational lifestyle intervention (the Healthy Eating and Lifestyle Programme (HELP)) on metabolic outcomes in obese adolescents: Findings from a randomized controlled trial. *Pediatric Diabetes.* 2015;16:45.
65. Christie D, Hudson L, Mathiot A, et al. Assessing the efficacy of the Healthy Eating and Lifestyle Programme (HELP) compared with enhanced standard care of the obese adolescent in the community: study protocol for a randomized controlled trial. *Trials.* 2011;12.
66. Christie D, Hudson LD, Kinra S, et al. A community-based motivational personalised lifestyle intervention to reduce BMI in obese adolescents: results from the Healthy Eating and Lifestyle Programme (HELP) randomised controlled trial. *Arch Dis Child.* 2017;102(8):695-701.
67. Christison AL, Evans TA, Bleess BB, Wang H, Aldag JC, Binns HJ. Exergaming for Health: A Randomized Study of Community-Based Exergaming Curriculum in Pediatric Weight Management. *Games Health J.* 2016;5(6):413-421.
68. Christison A, Khan HA. Exergaming for Health: A Community-Based Pediatric Weight Management Program Using Active Video Gaming. *Clin Pediatr.* 2012;51(4):382-388.
69. Cohen TR, Hazell TJ, Vanstone CA, Rodd C, Weiler HA. A family-centered lifestyle intervention for obese six- to eight-year-old children: Results from a one-year randomized controlled trial conducted in Montreal, Canada. *Canadian Journal of Public Health-Revue Canadienne De Sante Publique.* 2016;107(4-5):E453-E460.
70. Cohen TR, Hazell TJ, Vanstone CA, Plourde H, Rodd CJ, Weiler HA. A family-centered lifestyle intervention to improve body composition and bone mass in overweight and obese children 6 through 8 years: a randomized controlled trial study protocol. *BMC Public Health.* 2013;13:383.
71. Coppins DF, Margetts BM, Fa JL, Brown M, Garrett F, Huelin S. Effectiveness of a multi-disciplinary family-based programme for treating childhood obesity (the Family Project). *Eur J Clin Nutr.* 2011;65:903-909.
72. Crabtree VM, Moore JB, Jacks DE, Cerrito P, Topp RV. A transtheoretical, case management approach to the treatment of pediatric obesity. *J Prim Care Community Health.* 2010;1:4-7.
73. Croker H, Viner RM, Nicholls D, et al. Family-based behavioural treatment of childhood obesity in a UK National Health Service setting: randomized controlled trial. *Int J Obes Lond.* 2012;36:16-26.
74. Davis JN, Ventura EE, Alexander KE, et al. Feasibility of a home-based versus classroom-based nutrition intervention to reduce obesity and type 2 diabetes in Latino youth. *Int J Pediatr Obes.* 2007;2:22-30.
75. Davis JN, Ventura EE, Tung A, et al. Effects of a randomized maintenance intervention on adiposity and metabolic risk factors in overweight minority adolescents. *Pediatr Obes.* 2012;7:16-27.
76. Davis JN, Kelly LA, Lane CJ, et al. Randomized control trial to improve adiposity and insulin resistance in overweight Latino adolescents. *Obesity.* 2009;17(8):1542-1548.
77. Davis AM, James RL, Boles RE, Goetz JR, Belmont J, Malone B. The use of TeleMedicine in the treatment of paediatric obesity: feasibility and acceptability. *Matern Child Nutr.* 2011;7:71-79.
78. Davis AM, Sampilo M, Gallagher KS, Landrum Y, Malone B. Treating rural pediatric obesity through telemedicine: outcomes from a small randomized controlled trial. *J Pediatr Psychol.* 2013;38:932-943.
79. Davis AM, Stough CO, Black WR, et al. Outcomes of a weight management program conjointly addressing parent and child health. *Childrens Health Care.* 2016;45(2):227-240.
80. Davis AM, Sampilo M, Gallagher KS, et al. Treating rural paediatric obesity through telemedicine vs. telephone: Outcomes from a cluster randomized controlled trial. *J Telemed Telecare.* 2016;22(2):86-95.
81. Davoli AM, Broccoli S, Bonvicini L, et al. Pediatrician-led motivational interviewing to treat overweight children: an RCT. *Pediatrics.* 2013;132:e1236-1246.

82. De Bock F, Breitenstein L, Fischer JE. Positive impact of a pre-school-based nutritional intervention on children's fruit and vegetable intake: results of a cluster-randomized trial. *Public Health Nutr.* 2012;15:466-475.
83. de Niet J, Timman R, Bauer S, et al. Short message service reduces dropout in childhood obesity treatment: a randomized controlled trial. *Health Psychol.* 2012;31:797-805.
84. de Niet J, Timman R, Bauer S, et al. The effect of a short message service maintenance treatment on body mass index and psychological well-being in overweight and obese children: a randomized controlled trial. *Pediatr Obes.* 2012;7:205-219.
85. DeBar LL, Stevens VJ, Perrin N, et al. A primary care-based, multicomponent lifestyle intervention for overweight adolescent females. *Pediatrics.* 2012;129:e611-620.
86. Nystrom CD, Sandin S, Henriksson P, et al. Mobile-based intervention intended to stop obesity in preschool-aged children: the MINISTOP randomized controlled trial. *American Journal of Clinical Nutrition.* 2017;105(6):1327-1335.
87. Delisle C, Sandin S, Forsum E, et al. A web- and mobile phone-based intervention to prevent obesity in 4-year-olds (MINISTOP): a population-based randomized controlled trial. *BMC Public Health.* 2015;15:95.
88. Díaz RG, Esparza-Romero J, Moya-Camarena SY, Robles-Sardín AE, Valencia ME. Lifestyle intervention in primary care settings improves obesity parameters among Mexican youth. *J Am Diet Assoc.* 2010;110:285-290.
89. Doyle AC, Goldschmidt A, Huang C, Winzelberg AJ, Taylor CB, Wilfley DE. Reduction of overweight and eating disorder symptoms via the Internet in adolescents: A randomized controlled trial. *J Adoles Health* 2008;43:172-179.
90. Doyle-Baker PK, Venner AA, Lyon ME, Fung T. Impact of a combined diet and progressive exercise intervention for overweight and obese children: the B.E. H.I.P. study. *Appl Physiol Nutr Metab.* 2011;36:515-525.
91. Gillette MLD, Stough CO, Best CM, Beck AR, Hampl SE. Comparison of a Condensed 12-Week Version and a 24-Week Version of a Family-Based Pediatric Weight Management Program. *Childhood Obesity.* 2014;10(5):375-382.
92. Hampl S, Stough CO, Cordts KP, Best C, Blackburn K, Gillette MLD. Effectiveness of a Hospital-Based Multidisciplinary Pediatric Weight Management Program: Two-Year Outcomes of PHIT Kids. *Childhood Obesity.* 2016;12(1):20-25.
93. Duffy G, Spence SH. The effectiveness of cognitive self-management as an adjunct to a behavioural intervention for childhood obesity: a research note. *J Child Psychol Psychiatry.* 1993;34:1043-1050.
94. Duggins M, Cherven P, Carrithers J, Messamore J, Harvey A. Impact of family YMCA membership on childhood obesity: a randomized controlled effectiveness trial. *J Am Board Fam Med.* 2010;23:323-333.
95. Ebbeling CB, Feldman HA, Osganian SK, Chomitz VR, Ellenbogen SJ, Ludwig DS. Effects of decreasing sugar-sweetened beverage consumption on body weight in adolescents: a randomized, controlled pilot study. *Pediatrics.* 2006;117:673-680.
96. Ebbeling CB, Feldman HA, Chomitz VR, et al. A randomized trial of sugar-sweetened beverages and adolescent body weight. *N Engl J Med.* 2012;367:1407-1416.
97. Elder JP, Crespo NC, Corder K, et al. Childhood obesity prevention and control in city recreation centres and family homes: the MOVE/me Muevo Project. *Pediatr Obes.* 2013;9:218-231.
98. Epstein LH, McKenzie SJ, Valoski A, Klein KR, Wing RR. Effects of mastery criteria and contingent reinforcement for family-based child weight control. *Addict Behav.* 1994;19:135-145.
99. Epstein LH, Valoski AM, Vara LS, et al. Effects of decreasing sedentary behavior and increasing activity on weight change in obese children. *Health Psychol.* 1995;14:109-115.
100. Epstein LH, Paluch RA, Gordy CC, Dorn J. Decreasing sedentary behaviors in treating pediatric obesity. *Arch Pediatr Adolesc Med.* 2000;154:220-226.
101. Wrotniak BH, Epstein LH, Paluch RA, Roemmich JN. The relationship between parent and child self-reported adherence and weight loss. *Obes Res.* 2005;13:1089-1096.

102. Epstein LH, Paluch RA, Gordy CC, Saelens BE, Ernst MM. Problem solving in the treatment of childhood obesity. *J Consult Clin Psychol.* 2000;68:717-721.
103. Epstein LH, Gordy CC, Raynor HA, Beddome M, Kilanowski CK, Paluch R. Increasing fruit and vegetable intake and decreasing fat and sugar intake in families at risk for childhood obesity. *Obes Res.* 2001;9:171-178.
104. Epstein LH, Paluch RA, Raynor HA. Sex differences in obese children and siblings in family-based obesity treatment. *Obes Res.* 2001;9:746-753.
105. Epstein LH, Paluch RA, Kilanowski CK, Raynor HA. The effect of reinforcement or stimulus control to reduce sedentary behavior in the treatment of pediatric obesity. *Health Psychol.* 2004;23:371-380.
106. Epstein LH, Roemmich JN, Stein RI, Paluch RA, Kilanowski CK. The challenge of identifying behavioral alternatives to food: clinic and field studies. *Ann Behav Med.* 2005;30:201-209.
107. Epstein LH, Roemmich JN, Robinson JL, et al. A randomized trial of the effects of reducing television viewing and computer use on body mass index in young children. *Arch Pediatr Adolesc Med.* 2008;162:239-245.
108. Epstein LH, Paluch RA, Beecher MD, Roemmich JN. Increasing healthy eating vs. reducing high energy-dense foods to treat pediatric obesity. *Obesity (Silver Spring).* 2008;16:318-326.
109. Epstein LH, Paluch RA, Wrotniak BH, et al. Cost-effectiveness of family-based group treatment for child and parental obesity. *Child Obes.* 2014;10:114-121.
110. Epstein LH, Kilanowski C, Paluch RA, Raynor H, Daniel TO. Reducing variety enhances effectiveness of family-based treatment for pediatric obesity. *Eat Behav.* 2015;17:140-143.
111. Esfarjani F, Khalafi M, Mohammadi F, et al. Family-based intervention for controlling childhood obesity: an experience among Iranian children. *Int J Prev Med.* 2013;4:358-365.
112. Estabrooks PA, Shoup JA, Gattshall M, Dandamudi P, Shetterly S, Xu S. Automated telephone counseling for parents of overweight children: a randomized controlled trial. *Am J Prev Med.* 2009;36:35-42.
113. Falbe J, Cadiz AA, Tantoco NK, Thompson HR, Madsen KA. Active and Healthy Families: A Randomized Controlled Trial of a Culturally Tailored Obesity Intervention for Latino Children. *Academic pediatrics.* 2015;15(4):386-395.
114. Fitzgibbon ML, Stolley MR, Schiffer L, Van H, L, KauferChristoffel K, Dyer A. Two-year follow-up results for Hip-Hop to Health Jr.: a randomized controlled trial for overweight prevention in preschool minority children. *J Pediatr.* 2005;146:618-625.
115. Fitzgibbon ML, Stolley MR, Dyer AR, VanHorn L, KauferChristoffel K. A community-based obesity prevention program for minority children: rationale and study design for Hip-Hop to Health Jr. *Prev Med.* 2002;34(2):289-297.
116. Fitzgibbon ML, Stolley MR, Schiffer L, Horn L, KauferChristoffel K, Dyer A. Hip-Hop to Health Jr. for Latino Preschool Children. *Obesity (Silver Spring).* 2006;14(9):1616-1625.
117. Stolley MR, Fitzgibbon ML, Dyer A, Van H, L, KauferChristoffel K, Schiffer L. Hip-Hop to Health Jr., an obesity prevention program for minority preschool children: baseline characteristics of participants. *Prev Med.* 2003;36:320-329.
118. Fitzgibbon ML, Stolley MR, Schiffer L, et al. Family-based hip-hop to health: outcome results. *Obesity (Silver Spring).* 2013;21:274-283.
119. Flodmark CE, Ohlsson T, Ryden O, Sveger T. Prevention of progression to severe obesity in a group of obese schoolchildren treated with family therapy. *Pediatrics.* 1993;91:880-884.
120. Foley L, Jiang YN, Mhurchu CN, et al. The effect of active video games by ethnicity, sex and fitness: subgroup analysis from a randomised controlled trial. *International Journal of Behavioral Nutrition and Physical Activity.* 2014;11.
121. Maddison R, Foley L, Ni Mhurchu C, et al. Effects of active video games on body composition: a randomized controlled trial. *American Journal of Clinical Nutrition.* 2011;94(1):156-163.
122. Maddison R, Foley L, Mhurchu CN, et al. Feasibility, design and conduct of a pragmatic randomized controlled trial to reduce overweight and obesity in children: The electronic games to aid motivation to exercise (eGAME) study. *BMC Public Health.* 2009;9.
123. Ford AL, Bergh C, Sodersten P, et al. Treatment of childhood obesity by retraining eating behaviour: Randomised controlled trial. *BMJ.* 2010;340:b5388.

124. French SA, Mitchell NR, Hannan PJ. Decrease in television viewing predicts lower body mass index at 1-year follow-up in adolescents, but not adults. *J Nutr Educ Behav.* 2012;44:415-422.
125. French SA, Gerlach AF, Mitchell NR, Hannan PJ, Welsh EM. Household obesity prevention: Take Action--a group-randomized trial. *Obesity (Silver Spring).* 2011;19:2082-2088.
126. French SA, Sherwood NE, Jaka MM, Haapala JL, Ebbeling CB, Ludwig DS. Physical changes in the home environment to reduce television viewing and sugar-sweetened beverage consumption among 5-to 12-year-old children: a randomized pilot study. *Pediatr Obes.* 2016;11(5):E12-E15.
127. Frenn M, Pruszynski JE, Felzer H, Zhang J. Authoritative feeding behaviors to reduce child BMI through online interventions. *J Spec Pediatr Nurs.* 2013;18:65-77.
128. Fulkerson JA, Rydell S, Kubik MY, et al. Healthy Home Offerings via the Mealtime Environment (HOME): feasibility, acceptability, and outcomes of a pilot study. *Obesity (Silver Spring).* 2010;18 Suppl 1:S69-74.
129. Fulkerson JA, Friend S, Flattum C, et al. Promoting healthful family meals to prevent obesity: HOME Plus, a randomized controlled trial. *International Journal of Behavioral Nutrition and Physical Activity.* 2015;12.
130. Fulkerson JA, Neumark-Sztainer D, Story M, et al. The Healthy Home Offerings via the Mealtime Environment (HOME) Plus study: design and methods. *Contemp Clin Trials.* 2014;38(1):59-68.
131. Flattum C, Draxten M, Horning M, et al. HOME Plus: Program design and implementation of a family-focused, community-based intervention to promote the frequency and healthfulness of family meals, reduce children's sedentary behavior, and prevent obesity. *Int J Behav Nutr Phys Act.* 2015;12:53.
132. Garipagaoglu M, Sahip Y, Darendeliler F, Akdikmen O, Kopuz S, Sut N. Family-based group treatment versus individual treatment in the management of childhood obesity: randomized, prospective clinical trial. *Eur J Pediatr.* 2009;168:1091-1099.
133. Gerards SM, Dagnelie PC, Jansen MW, et al. Lifestyle Triple P: a parenting intervention for childhood obesity. *BMC Public Health.* 2012;12:267.
134. Gerards SM, Dagnelie PC, Gubbels JS, et al. The effectiveness of Lifestyle Triple P in the Netherlands: a randomized controlled trial. *PLoS One.* 2015;10(4):e0122240.
135. Ghatrehsamani S, Khavarian N, Beizaei M, Ramedan R, Poursafa P, Kelishadi R. Effect of different physical activity training methods on overweight adolescents. *ARYA Atheroscler.* 2010;6:45-49.
136. Gillis D, Brauner M, Granot E. A community-based behavior modification intervention for childhood obesity. *J Pediatr Endocrinol Metab.* 2007;20:197-203.
137. Golan M, Weizman A, Apter A, Fainaru M. Parents as the exclusive agents of change in the treatment of childhood obesity. *Am J Clin Nutr.* 1998;67:1130-1135.
138. Golan M, Fainaru M, Weizman A. Role of behaviour modification in the treatment of childhood obesity with the parents as the exclusive agents of change. *Int J Obes Relat Metab Disord.* 1998;22:1217-1224.
139. Golan M, Crow S. Targeting parents exclusively in the treatment of childhood obesity: long-term results. *Obes Res.* 2004;12:357-361.
140. Golan M, Kaufman V, Shahar DR. Childhood obesity treatment: targeting parents exclusively v. parents and children. *Br J Nutr.* 2006;95:1008-1015.
141. Goldfield GS, Epstein LH, Kilanowski CK, Paluch RA, Kogut-Bossler B. Cost-effectiveness of group and mixed family-based treatment for childhood obesity. *Int J Obes Relat Metab Disord.* 2001;25:1843-1849.
142. Golley RK, Magarey AM, Baur LA, Steinbeck KS, Daniels LA. Twelve-month effectiveness of a parent-led, family-focused weight-management program for prepubertal children: a randomized, controlled trial. *Pediatrics.* 2007;119:517-525.
143. Golley RK, Magarey AM, Daniels LA. Children's food and activity patterns following a six-month child weight management program. *Int J Pediatr Obes.* 2011;6:409-414.
144. Gourlan M, Sarrazin P, Trouilloud D. Motivational interviewing as a way to promote physical activity in obese adolescents: a randomised-controlled trial using self-determination theory as an explanatory framework. *Psychol Health.* 2013;28:1265-1286.
145. Gunnarsdottir T, Sigurdardottir ZG, Njardvik U, Olafsdottir AS, Bjarnason R. A randomized-controlled pilot study of Epstein's family-based behavioural treatment for childhood obesity in a clinical setting in Iceland. *Nordic Psychology.* 2011;63:6-19.

146. Guo H, Zeng X, Zhuang Q, Zheng Y, Chen S. Intervention of childhood and adolescents obesity in Shantou city. *Obes Res Clin Pract.* 2015;9(4):357-364.
147. Habib-Mourad C, Ghandour LA, Moore HJ, et al. Promoting healthy eating and physical activity among school children: findings from Health-E-PALS, the first pilot intervention from Lebanon. *BMC Public Health.* 2014;14.
148. Habib-Mourad CaM, H. and Nabhani, Z.M. and Hwalla, N. and Summerbell, C. Health-E-PALS : promoting healthy eating and physical activity in Lebanese school children – intervention development. *Education and Health.* 2014;32(1):3-8.
149. Haines J, Rifas-Shiman SL, Gross D, McDonald J, Kleinman K, Gillman MW. Randomized trial of a prevention intervention that embeds weight-related messages within a general parenting program. *Obesity (Silver Spring).* 2016;24(1):191-199.
150. Haire-Joshu D, Nanney MS, Elliott M, et al. The use of mentoring programs to improve energy balance behaviors in high-risk children. *Obesity (Silver Spring).* 2010;18 Suppl 1:S75-83.
151. Harder-Lauridsen NM, Birk NM, Ried-Larsen M, et al. A randomized controlled trial on a multicomponent intervention for overweight school-aged children - Copenhagen, Denmark. *BMC Pediatr.* 2014;14.
152. Golley RK, Hendrie GA. The impact of replacing regular- with reduced-fat dairy foods on children's wider food intake: secondary analysis of a cluster RCT. *Eur J Clin Nutr.* 2012;66:1130-1134.
153. Hendrie GA, Golley RK. Changing from regular-fat to low-fat dairy foods reduces saturated fat intake but not energy intake in 4-13-y-old children. *Am J Clin Nutr.* 2011;93:1117-1127.
154. Herget S, Reichardt S, Grimm A, et al. High-Intensity Interval Training for Overweight Adolescents: Program Acceptance of a Media Supported Intervention and Changes in Body Composition. *Int J Environ Res Public Health.* 2016;13(11).
155. Bluher S, Kapplinger J, Herget S, et al. Cardiometabolic risk markers, adipocyte fatty acid binding protein (aFABP) and the impact of high-intensity interval training (HIIT) in obese adolescents. *Metabolism.* 2017;68:77-87.
156. Hidayanty H, Bardosono S, Khusun H, Damayanti R, Kolopaking R. A social cognitive theory-based programme for eating patterns and sedentary activity among overweight adolescents in Makassar, South Sulawesi: a cluster randomised controlled trial. *Asia Pacific Journal of Clinical Nutrition.* 2016;25:S83-S92.
157. Ho J, Pedersen SD, Virtanen H, Nettel-Aguirre A, Huang C. Family Intervention for Obese/Overweight Children Using Portion Control Strategy (FOCUS) for Weight Control: A Randomized Controlled Trial. *Glob Pediatr Health.* 2016;3:2333794x16669014.
158. Hofsteenge GH, Weijs PJ, Delemarre-van de Waal HA, de Wit M, Chinapaw MJ. Effect of the Go4it multidisciplinary group treatment for obese adolescents on health related quality of life: a randomised controlled trial. *BMC Public Health.* 2013;13:939.
159. Hofsteenge GH, Chinapaw MJ, Delemarre-van de Waal HA, Weijs PJ. Long-term effect of the Go4it group treatment for obese adolescents: A randomised controlled trial. *Clin Nutr.* 2014;33:385-391.
160. Hofsteenge GH, Chinapaw MJ, Weijs PJ, van Tulder MW, Delemarre-van de Waal HA. Go4it; study design of a randomised controlled trial and economic evaluation of a multidisciplinary group intervention for obese adolescents for prevention of diabetes mellitus type 2. *BMC Public Health.* 2008;8:410.
161. Hull PC, Buchowski M, Canedo JR, et al. Childhood obesity prevention cluster randomized trial for Hispanic families: outcomes of the healthy families study. *Pediatr Obes.* 2016.
162. Zoorob R, Buchowski MS, Beech BM, et al. Healthy families study: design of a childhood obesity prevention trial for Hispanic families. *Contemp Clin Trials.* 2013;35(2):108-121.
163. Hystad HT, Steinsbekk S, Ødegård R, Wichstrøm L, Gudbrandsen OA. A randomised study on the effectiveness of therapist-led v. self-help parental intervention for treating childhood obesity. *Br J Nutr.* 2013;110:1143-1150.
164. Israel AC, Guile CA, Baker JE, Silverman WK. An evaluation of enhanced self-regulation training in the treatment of childhood obesity. *J Pediatr Psychol.* 1994;19:737-749.
165. Janicke DM, Sallinen BJ, Perri MG, et al. Sensible treatment of obesity in rural youth (STORY): design and methods. *Contemp Clin Trials.* 2008;29:270-280.

166. Janicke DM, Sallinen BJ, Perri MG, et al. Comparison of parent-only vs family-based interventions for overweight children in underserved rural settings: outcomes from project STORY. *Arch Pediatr Adolesc Med.* 2008;162:1119-1125.
167. Janicke DM, Sallinen BJ, Perri MG, Lutes LD, Silverstein JH, Brumback B. Comparison of program costs for parent-only and family-based interventions for pediatric obesity in medically underserved rural settings. *J Rural Health.* 2009;25:326-330.
168. Jansen E, Mulkens S, Jansen A. Tackling childhood overweight: treating parents exclusively is effective. *Int J Obes Lond.* 2011;35:501-509.
169. Mehlenbeck RS, Jelalian E, Lloyd-Richardson EE, Hart CN. Effects of Behavioral Weight Control Intervention on Binge Eating Symptoms Among Overweight Adolescents. *Psychol Sch.* 2009;46:776-786.
170. Jelalian E, Hart CN, Mehlenbeck RS, et al. Predictors of attrition and weight loss in an adolescent weight control program. *Obesity (Silver Spring).* 2008;16:1318-1323.
171. Jelalian E, Mehlenbeck R, Lloyd-Richardson EE, Birmaher V, Wing RR. 'Adventure therapy' combined with cognitive-behavioral treatment for overweight adolescents. *Int J Obes (Lond).* 2006;30:31-39.
172. Lloyd-Richardson EE, Jelalian E, Sato AF, Hart CN, Mehlenbeck R, Wing RR. Two-year follow-up of an adolescent behavioral weight control intervention. *Pediatrics.* 2013;130:e281-e288.
173. Jelalian E, Sato A, Hart CN. The effect of group-based weight control intervention on adolescent psychosocial outcomes: Perceived peer rejection, social anxiety and self-concept. *Child Health Care.* 2011;40:197-211.
174. Jelalian E, Lloyd-Richardson EE, Mehlenbeck RS, et al. Behavioral weight control treatment with supervised exercise or peer-enhanced adventure for overweight adolescents. *J Pediatr.* 2010;157:923-928.
175. Sato AF, Jelalian E, Hart CN, et al. Associations between parent behavior and adolescent weight control. *J Pediatr Psychol.* 2011;36:451-460.
176. Jensen ME, Gibson PG, Collins CE, Hilton JM, Wood LG. Diet-induced weight loss in obese children with asthma: a randomized controlled trial. *Clin Exp Allergy.* 2013;43:775-784.
177. Jiang JX, Xia XL, Greiner T, Lian GL, Rosenqvist U. A two year family based behaviour treatment for obese children. *Arch Dis Child.* 2005;90:1235-1238.
178. Johnson WG, Hinkle LK, Carr RE, et al. Dietary and exercise interventions for juvenile obesity: long-term effect of behavioral and public health models. *Obes Res.* 1997;5:257-261.
179. Burrows T, Janet WM, Collins CE. Long-term changes in food consumption trends in overweight children in the HIKCUPS intervention. *J Pediatr Gastroenterol Nutr.* 2011;53:543-547.
180. Cliff DP, Okely AD, Morgan PJ, et al. Movement skills and physical activity in obese children: randomized controlled trial. *Med Sci Sports Exerc.* 2011;43:90-100.
181. Collins CE, Okely AD, Morgan PJ, et al. Parent diet modification, child activity, or both in obese children: an RCT. *Pediatrics.* 2011;127:619-627.
182. Okely AD, Collins CE, Morgan PJ, et al. Multi-site randomized controlled trial of a child-centered physical activity program, a parent-centered dietary-modification program, or both in overweight children: the HIKCUPS study. *J Pediatr.* 2010;157:388-394, 394.
183. Burrows T, Warren J, Collins C. The impact of a child obesity treatment intervention on parent child-feeding practices. *Int J Pediatr Obes.* 2010;5(1):43-50.
184. Burrows T, Warren JM, Baur LA, Collins CE. Impact of a child obesity intervention on dietary intake and behaviors. *Int J Obes (Lond).* 2008;32:1481-1488.
185. Jones RA, Warren JM, Okely AD, et al. Process evaluation of the Hunter Illawarra Kids Challenge Using Parent Support study: a multisite randomized controlled trial for the management of child obesity. *Health Promot Pract.* 2010;11:917-927.
186. Jones RA, Okely AD, Collins CE, et al. The HIKCUPS trial: a multi-site randomized controlled trial of a combined physical activity skill-development and dietary modification program in overweight and obese children. *BMC Public Health.* 2007;7:15.
187. Jones M, Luce KH, Osborne MI, et al. Randomized, controlled trial of an Internet-facilitated intervention for reducing binge eating and overweight in adolescents. *Pediatrics.* 2008;121:453-462.

188. Jones RA, Kelly J, Cliff DP, Batterham M, Okely AD. Acceptability and Potential Efficacy of Single-Sex After-School Activity Programs for Overweight and At-Risk Children: The Wollongong SPORT RCT. *Pediatr Exerc Sci.* 2015;27(4):535-545.
189. Kalarchian MA, Levine MD, Arslanian SA, et al. Family-based treatment of severe pediatric obesity: randomized, controlled trial. *Pediatrics.* 2009;124:1060-1068.
190. Wildes JE, Marcus MD, Kalarchian MA, Levine MD, Houck PR, Cheng Y. Self-reported binge eating in severe pediatric obesity: Impact on weight change in a randomized controlled trial of family-based treatment. *Int J Obes (Lond).* 2010;34:1143-1148.
191. Kalavainen M, Utriainen P, Vanninen E, Korppi M, Nuutinen O. Impact of childhood obesity treatment on body composition and metabolic profile. *World J Pediatr.* 2012;8:31-37.
192. Kalavainen M, Korppi M, Nuutinen O. Long-term efficacy of group-based treatment for childhood obesity compared with routinely given individual counselling. *Int J Obes (Lond).* 2011;35:530-533.
193. Kalavainen M, Karjalainen S, Martikainen J, Korppi M, Linnosmaa I, Nuutinen O. Cost-effectiveness of routine and group programs for treatment of obese children. *Pediatr Int.* 2009;51:606-611.
194. Kalavainen MP, Korppi MO, Nuutinen OM. Clinical efficacy of group-based treatment for childhood obesity compared with routinely given individual counseling. *Int J Obes (Lond).* 2007;31:1500-1508.
195. Kelishadi R, Hashemipour M, Mohammadifard N, Alikhassy H, Adeli K. Short- and long-term relationships of serum ghrelin with changes in body composition and the metabolic syndrome in prepubescent obese children following two different weight loss programmes. *Clin Endocrinol (Oxf).* 2008;69:721-729.
196. Kelishadi R, Hashemi M, Mohammadifard N, Asgary S, Khavarian N. Association of changes in oxidative and proinflammatory states with changes in vascular function after a lifestyle modification trial among obese children. *Clin Chem.* 2008;54:147-153.
197. Khadilkar VV, Pandit DS, Khadilkar AV, Chiplonkar SA, Kinare AS. Diet and exercise intervention, with special reference to micronutrients, reduces cardiometabolic risk in overweight children. *Indian J Endocrinol Metab.* 2012;16:124-133.
198. Khanal S, Welsby D, Lloyd B, Innes-Hughes C, Lukeis S, Rissel C. Effectiveness of a once per week delivery of a family-based childhood obesity intervention: a cluster randomised controlled trial. *Pediatr Obes.* 2016;11(6):475-483.
199. Kim HS, Park J, Park KY, Lee MN, Ham OK. Parent Involvement Intervention in Developing Weight Management Skills for both Parents and Overweight/Obese Children. *Asian Nurs Res.* 2016;10(1):11-17.
200. Kitzman-Ulrich H, Hampson R, Wilson DK, Presnell K, Brown A, O'Boyle M. An adolescent weight-loss program integrating family variables reduces energy intake. *J Am Diet Assoc.* 2009;109:491-496.
201. Klesges RC, Obarzanek E, Kumanyika S, et al. The Memphis Girls' health Enrichment Multi-site Studies (GEMS): an evaluation of the efficacy of a 2-year obesity prevention program in African American girls. *Arch Pediatr Adolesc Med.* 2010;164:1007-1014.
202. Stockton MB, McClanahan BS, Lanctot JQ, Klesges RC, Beech BM. Identification of facilitators and barriers to participation in weight gain prevention research by African American girls. *Contemp Clin Trials.* 2012;33:38-45.
203. Klesges RC, Obarzanek E, Klesges LM, et al. Memphis Girls health Enrichment Multi-site Studies (GEMS): Phase 2: design and baseline. *Contemp Clin Trials.* 2008;29:42-55.
204. Kobel S, Lammle C, Wartha O, Kesztyus D, Wirt T, Steinacker JM. Effects of a Randomised Controlled School-Based Health Promotion Intervention on Obesity Related Behavioural Outcomes of Children with Migration Background. *J Immigr Minor Healt.* 2017;19(2):254-262.
205. Kobel S, Wirt T, Schreiber A, et al. Intervention effects of a school-based health promotion programme on obesity related behavioural outcomes. *J Obes.* 2014;2014:476230.
206. Dreyhaupt J, Koch B, Wirt T, et al. Evaluation of a health promotion program in children: Study protocol and design of the cluster-randomized Baden-Wurttemberg primary school study [DRKS-ID: DRKS00000494]. *BMC Public Health.* 2012;12:157.
207. Kulendran M, King D, Schmidtke KA, et al. The use of commitment techniques to support weight loss maintenance in obese adolescents. *Psychology & Health.* 2016;31(11):1332-1341.

208. Kulik N, Ennett ST, Ward DS, Bowling JM, Fisher EB, Tate DF. Brief report: A randomized controlled trial examining peer support and behavioral weight loss treatment. *J Adolescence.* 2015;44:117-123.
209. Kulik NL, Fisher EB, Ward DS, Ennett ST, Bowling JM, Tate DF. Peer Support Enhanced Social Support in Adolescent Females During Weight Loss. *Am J Health Behav.* 2014;38(5):789-800.
210. Larsen LM, Hertel NT, Molgaard C, Christensen RD, Husby S, Jarbol DE. Early intervention for childhood overweight: A randomized trial in general practice. *Scand J Prim Health.* 2015;33(3):184-190.
211. Leach RA, Yates JM. Nutrition and youth soccer for childhood overweight: a pilot novel chiropractic health education intervention. *J Manipulative Physiol Ther.* 2008;31:434-441.
212. Leme ACB, Lubans DR, Guerra PH, Dewar D, Toassa EC, Philippi ST. Preventing obesity among Brazilian adolescent girls: Six-month outcomes of the Healthy Habits, Healthy Girls-Brazil school-based randomized controlled trial. *Prev Med.* 2016;86:77-83.
213. Leme ACB, Philippi ST. The "Healthy Habits, Healthy Girls" randomized controlled trial for girls: study design, protocol, and baseline results. *Cadernos De Saude Publica.* 2015;31(7):1381-1394.
214. Lochrie AS, Wysocki T, Hossain J, et al. The effects of a family-based intervention (FBI) for overweight/obese children on health and psychological functioning. *Clinical Practice in Pediatric Psychology.* 2013;1:159-170.
215. Looney SM, Raynor HA. Examining the Effect of Three Low-Intensity Pediatric Obesity Interventions: A Pilot Randomized Controlled Trial. *Clin Pediatr.* 2014;53(14):1367-1374.
216. Love-Osborne K, Fortune R, Sheeder J, Federico S, Haemer MA. School-Based Health Center-Based Treatment for Obese Adolescents: Feasibility and Body Mass Index Effects. *Childhood Obesity.* 2014;10(5):424-431.
217. Lubans DR, Smith JJ, Plotnikoff RC, et al. Assessing the sustained impact of a school-based obesity prevention program for adolescent boys: the ATLAS cluster randomized controlled trial. *International Journal of Behavioral Nutrition and Physical Activity.* 2016;13.
218. Smith JJ, Morgan PJ, Plotnikoff RC, et al. Rationale and study protocol for the 'Active Teen Leaders Avoiding Screen-time' (ATLAS) group randomized controlled trial: An obesity prevention intervention for adolescent boys from schools in low-income communities. *Contemp Clin Trials.* 2014;37(1):106-119.
219. Smith JJ, Morgan PJ, Plotnikoff RC, et al. Smart-Phone Obesity Prevention Trial for Adolescent Boys in Low-Income Communities: The ATLAS RCT. *Pediatrics.* 2014;134(3):E723-E731.
220. Luszczynska A, Horodyska K, Zarychta K, Liszewska N, Knoll N, Scholz U. Planning and self-efficacy interventions encouraging replacing energy-dense foods intake with fruit and vegetable: A longitudinal experimental study. *Psychology & Health.* 2016;31(1):40-64.
221. Macdonell K, Brogan K, Naar-King S, Ellis D, Marshall S. A pilot study of motivational interviewing targeting weight-related behaviors in overweight or obese African American adolescents. *J Adolesc Health.* 2012;50:201-203.
222. Macias-Cervantes MH, Malacara JM, Garay-Sevilla ME, Diaz-Cisneros FJ. Effect of recreational physical activity on insulin levels in Mexican/Hispanic children. *Eur J Pediatr.* 2009;168(10):1195-1202.
223. Maddison R, Mhurchu CN, Foley L, et al. Screen-time Weight-loss Intervention Targeting Children at Home (SWITCH): A randomized controlled trial study protocol. *BMC Public Health.* 2011;11(1):1.
224. Maddison R, Marsh S, Foley L, et al. Screen-time Weight-loss Intervention Targeting Children at Home (SWITCH): a randomized controlled trial. *Int J Behav Nutr Phys Act.* 2014;11(1):1-11.
225. Madsen K, Thompson H, Adkins A, Crawford Y. School-community partnerships: a cluster-randomized trial of an after-school soccer program. *JAMA Pediatr.* 2013;167:321-326.
226. Madsen K, Linchey J, Gerstein D, et al. Energy Balance 4 Kids with Play: Results from a Two-Year Cluster-Randomized Trial. *Childhood Obesity.* 2015;11(4):375-383.
227. Myers EF, Gerstein DE, Foster J, et al. Energy Balance for Kids with Play: Design and Implementation of a Multi-Component School-Based Obesity Prevention Program. *Childhood Obesity.* 2014;10(3):251-259.
228. Magarey AM, Perry RA, Baur LA, et al. A parent-led family-focused treatment program for overweight children aged 5 to 9 years: The PEACH RCT. *Pediatrics.* 2011;127:214-222.
229. Majumdar I, Bethin K, Quattrin T. Weight trajectory of youth with new-onset type 1 diabetes comparing standard and enhanced dietary education. *Endocrine.* 2015;49(1):155-162.

230. Markert J, Alff F, Zschaler S, Gausche R, Kiess W, Blher S. Prevention of childhood obesity: recruiting strategies via local paediatricians and study protocol for a telephone-based counselling programme. *Obes Res Clin Pract.* 2013;7:e476-486.
231. Markert J, Herget S, Petroff D, et al. Telephone-based adiposity prevention for families with overweight children (TAFF-study): One year outcome of a randomized, controlled trial. *Int J Environ Res Public Health.* 2014;11(10):10327-10344.
232. Salcedo Aguilar F, Martinez-Vizcaíno V, Sanchez López M, et al. Impact of an after-school physical activity program on obesity in children. *J Pediatr.* 2010;157:36-42.
233. Martínez Vizcaíno V, Salcedo Aguilar F, Franquelo Gutíerrez R, et al. Assessment of an after-school physical activity program to prevent obesity among 9- to 10-year-old children: a cluster randomized trial. *Int J Obes (Lond).* 2008;32:12-22.
234. Martínez-Andrade GO, Cespedes EM, Rifas-Shiman SL, et al. Feasibility and impact of Creciendo Sanos, a clinic-based pilot intervention to prevent obesity among preschool children in Mexico City. *BMC Pediatr.* 2014;14:77.
235. Martínez-Vizcaíno V S-LM, Salcedo-Aguilar F, Notario-Pacheco B, Solera-Martínez M, Moya-Martínez P, Franquelo-Morales P, López-Martínez S, Rodríguez-Artalejo F, group. MOVI-. Protocol of a randomized cluster trial to assess the effectiveness of the MOVI-2 program on overweight prevention in schoolchildren. *Rev Esp Cardiol (Engl Ed).* 2012;65:427-433.
236. Martínez-Vizcaíno V, Sánchez-López M, Notario-Pacheco B, et al. Gender differences on effectiveness of a school-based physical activity intervention for reducing cardiometabolic risk: a cluster randomized trial. *Int J Behav Nutr Phys Act* 2014;11(1):154.
237. McCallum Z, Wake M, Gerner B, et al. Outcome data from the LEAP (Live, Eat and Play) trial: a randomized controlled trial of a primary care intervention for childhood overweight/mild obesity. *Int J Obes (Lond).* 2007;31:630-636.
238. McCallum Z, Wake M, Gerner B, et al. Can Australian general practitioners tackle childhood overweight/obesity? Methods and processes from the LEAP (Live, Eat and Play) randomized controlled trial. *J Paediatr Child Health.* 2005;41:488-494.
239. Wake M, Gold L, McCallum Z, Gerner B, Waters E. Economic evaluation of a primary care trial to reduce weight gain in overweight/obese children: the LEAP trial. *Ambul Pediatr.* 2008;8:336-341.
240. Melnyk BM, Jacobson D, Kelly SA, et al. Twelve-Month Effects of the COPE Healthy Lifestyles TEEN Program on Overweight and Depressive Symptoms in High School Adolescents. *J School Health.* 2015;85(12):861-870.
241. Melnyk BM, Small L, Morrison-Beedy D, et al. The COPE Healthy Lifestyles TEEN program: feasibility, preliminary efficacy, & lessons learned from an after school group intervention with overweight adolescents. *J Pediatr Health Care.* 2007;21:315-322.
242. Mendoza JA, Baranowski T, Jaramillo S, et al. Fit 5 Kids TV Reduction Program for Latino Preschoolers A Cluster Randomized Controlled Trial. *Am J Prev Med.* 2016;50(5):584-592.
243. Moens E, Braet C. Training parents of overweight children in parenting skills: a 12-month evaluation. *Behav Cogn Psychother.* 2012;40:1-18.
244. Burrows T, Morgan PJ, Lubans DR, et al. Dietary outcomes of the healthy dads healthy kids randomised controlled trial. *J Pediatr Gastroenterol Nutr.* 2012;55:408-411.
245. Lubans DR, Morgan PJ, Collins CE, Okely AD, Burrows T, Callister R. Mediators of weight loss in the 'Healthy Dads, Healthy Kids' pilot study for overweight fathers. *Int J Behav Nutr Phys Act.* 2012;9:45.
246. Morgan PJ, Lubans DR, Callister R, et al. The 'Healthy Dads, Healthy Kids' randomized controlled trial: efficacy of a healthy lifestyle program for overweight fathers and their children. *Int J Obes (Lond).* 2011;35:436-447.
247. Munsch S, Roth B, Michael T, et al. Randomized controlled comparison of two cognitive behavioral therapies for obese children: mother versus mother-child cognitive behavioral therapy. *Psychother Psychosom.* 2008;77:235-246.
248. Naar-King S, Ellis D, Kolmodin K, et al. A randomized pilot study of multisystemic therapy targeting obesity in African-American adolescents. *J Adolesc Health.* 2009;45:417-419.

249. MacDonell K, Ellis D, Naar-King S, Cunningham P. Predictors of home-based obesity treatment efficacy for African American youth. *Child.* 2010;39:1-14.
250. Nemet D, Berger-Shemesh E, Wolach B, Eliakim A. A combined dietary-physical activity intervention affects bone strength in obese children and adolescents. *Int J Sports Med.* 2006;27:666-671.
251. Nemet D, Barzilay-Teeni N, Eliakim A. Treatment of childhood obesity in obese families. *J Pediatr Endocrinol Metab.* 2008;21:461-467.
252. Nemet D, Oren S, Pantanowitz M, Eliakim A. Effects of a multidisciplinary childhood obesity treatment intervention on adipocytokines, inflammatory and growth mediators. *Horm Res Paediatr.* 2013;79:325-332.
253. Nemet D, Ben-Haim I, Pantanowits M, Eliakim A. Effects of a combined intervention for treating severely obese prepubertal children. *J Pediatr Endocrinol Metab.* 2013;26:91-96.
254. Neumark-Sztainer D, Haines J, Robinson-O'Brien R, et al. 'Ready. Set. ACTION!' A theater-based obesity prevention program for children: a feasibility study. *Health Educ Res.* 2009;24:407-420.
255. Nollen NL, Mayo MS, Carlson SE, Rapoff MA, Goggin KJ, Ellerbeck EF. Mobile technology for obesity prevention: a randomized pilot study in racial- and ethnic-minority girls. *Am J Prev Med.* 2014;46:404-408.
256. Nourian M, Kelishadi R, Najimi A. Lifestyle Interventions and Weight Control of Adolescents With Abdominal Obesity: A Randomized Controlled Trial Based on Health Belief Model. *Iranian Red Crescent Medical Journal.* 2017;19(2).
257. Nova A, Russo A, Sala E. Long-term management of obesity in paediatric office practice: experimental evaluation of two different types of intervention. *Ambulatory Child Health* 2001;7:239-247.
258. Novotny R, Nigg CR, Li FF, Wilkens LR. Pacific Kids DASH for Health (PacDASH) Randomized, Controlled Trial with DASH Eating Plan Plus Physical Activity Improves Fruit and Vegetable Intake and Diastolic Blood Pressure in Children. *Childhood Obesity.* 2015;11(2):177-186.
259. Nyberg G, Norman A, Sundblom E, Zeebari Z, Elinder LS. Effectiveness of a universal parental support programme to promote health behaviours and prevent overweight and obesity in 6-year-old children in disadvantaged areas, the Healthy School Start Study II, a cluster-randomised controlled trial. *International Journal of Behavioral Nutrition and Physical Activity.* 2016;13.
260. Ledoux T, Hilmers A, Watson K, Baranowski T, O'Connor TM. Development and feasibility of an objective measure of patient-centered communication fidelity in a pediatric obesity intervention. *J Nutr Educ Behav.* 2013;45:349-354.
261. O'Connor TM, Hilmers A, Watson K, Baranowski T, Giardino AP. Feasibility of an obesity intervention for paediatric primary care targeting parenting and children: Helping HAND. *Child Care Health Dev.* 2013;39:141-149.
262. Ostbye T, Krause KM, Stroo M, et al. Parent-focused change to prevent obesity in preschoolers: results from the KAN-DO study. *Prev Med.* 2012;55:188-195.
263. Ostbye T, Zucker NL, Krause KM, et al. Kids and adults now! Defeat Obesity (KAN-DO): rationale, design and baseline characteristics. *Contemp Clin Trials.* 2011;32:461-469.
264. Paineau DL, Beaufils F, Boulier A, et al. Family dietary coaching to improve nutritional intakes and body weight control: a randomized controlled trial. *Arch Pediatr Adolesc Med.* 2008;162:34-43.
265. Pakpour AH, Gellert P, Dombrowski SU, Fridlund B. Motivational Interviewing With Parents for Obesity: An RCT. *Pediatrics.* 2015;135(3):E644-E652.
266. Papadaki A, Linardakis M, Larsen TM, et al. The effect of protein and glycemic index on children's body composition: the DiOGenes randomized study. *Pediatrics.* 2010;126:e1143-1152.
267. Larsen TM, Dalskov S, Van Baak M, et al. The Diet, Obesity and Genes (Diogenes) Dietary Study in eight European countries–a comprehensive design for long-term intervention. *Obesity Reviews.* 2010;11(1):76-91.
268. Parra-Medina D, Mojica C, Liang YY, Ouyang YJ, Ramos AI, Gomez I. Promoting Weight Maintenance among Overweight and Obese Hispanic Children in a Rural Practice. *Childhood Obesity.* 2015;11(4):355-363.
269. Patrick K, Calfas KJ, Norman GJ, et al. Randomized controlled trial of a primary care and home-base intervention for physical activity and nutrition behaviors: PACE+ for adolescents. *Arch Pediatr Adolesc Med.* 2006;160:128-136.

270. Patrick K, Norman GJ, Davila EP, et al. Outcomes of a 12-month technology-based intervention to promote weight loss in adolescents at risk for type 2 diabetes. *J Diabetes Sci Technol.* 2013;7:759-770.
271. Pbert L, Druker S, Barton B, et al. A School-Based Program for Overweight and Obese Adolescents: A Randomized Controlled Trial. *J School Health.* 2016;86(10):699-708.
272. Pedrosa C, Oliveira BM, Albuquerque I, Simões-Pereira C, Vaz-de-Almeida MD, Correia F. Markers of metabolic syndrome in obese children before and after 1-year lifestyle intervention program. *Eur J Nutr.* 2011;50:391-400.
273. Pedrosa C, Oliveira BM, Albuquerque I, Simões-Pereira C, Vaz-de-Almeida MD, Correia F. Metabolic syndrome, adipokines and ghrelin in overweight and obese schoolchildren: results of a 1-year lifestyle intervention programme. *Eur J Pediatr.* 2011;170:483-492.
274. Quattrin T, Roemmich JN, Paluch R, Yu J, Epstein LH, Ecker MA. Efficacy of family-based weight control program for preschool children in primary care. *Pediatrics.* 2012;130:660-666.
275. Quattrin T, Roemmich JN, Paluch R, Jihnhee Y, Epstein LH, Ecker MA. Treatment Outcomes of Overweight Children and Parents in the Medical Home. *Pediatrics.* 2014;134(2):290-297.
276. Raynor HA, Kilanowski CK, Esterlis I, Epstein LH. A cost-analysis of adopting a healthful diet in a family-based obesity treatment program. *J Am Diet Assoc.* 2002;102:645-656.
277. Raynor HA, Osterholt KM, Hart CN, Jelalian E, Vivier P, Wing RR. Efficacy of U.S. paediatric obesity primary care guidelines: two randomized trials. *Pediatr Obes.* 2012;7:28-38.
278. Reinehr T, Schaefer A, Winkel K, Finne E, Toschke AM, Kolip P. An effective lifestyle intervention in overweight children: findings from a randomized controlled trial on "Obeldicks light". *Clin Nutr.* 2010;29:331-336.
279. Schaefer A, Winkel K, Finne E, Kolip P, Reinehr T. An effective lifestyle intervention in overweight children: one-year follow-up after the randomized controlled trial on Obeldicks light'.'. *Clin Nutr.* 2011;30:629-633.
280. Reinehr T, Schaefer A, Winkel K, Finne E, Kolip P. Development and evaluation of the lifestyle intervention 'Obeldicks light' for overweight children and adolescents. *J Public Health.* 2011;19:377-384.
281. Rerksuppaphol L, Rerksuppaphol S. Internet Based Obesity Prevention Program for Thai School Children- A Randomized Control Trial. *Journal of Clinical and Diagnostic Research.* 2017;11(3):Sc7-Sc11.
282. Resnick EA, Bishop M, O'Connell A, et al. The CHEER study to reduce BMI in Elementary School students: a school-based, parent-directed study in Framingham, Massachusetts. *J Sch Nurs.* 2009;25:361-372.
283. Resnicow K, Taylor R, Baskin M, McCarty F. Results of go girls: a weight control program for overweight African-American adolescent females. *Obes Res.* 2005;13:1739-1748.
284. Resnicow K, McMaster F, Woolford S, et al. Study design and baseline description of the BMI2 trial: reducing paediatric obesity in primary care practices. *Pediatr Obes.* 2012;7:3-15.
285. Resnicow K, McMaster F, Bocian A, et al. Motivational interviewing and dietary counseling for obesity in primary care: an RCT. *Pediatrics.* 2015;135(4):649-657.
286. Robertson W, Fleming J, Kamal A, et al. Randomised controlled trial evaluating the effectiveness and cost-effectiveness of 'Families for Health', a family-based childhood obesity treatment intervention delivered in a community setting for ages 6 to 11 years. *Health Technol Asses.* 2017;21(1):1-+.
287. Robertson W, Fleming J, Kamal A, et al. Randomised controlled trial and economic evaluation of the 'Families for Health' programme to reduce obesity in children. *Arch Dis Child.* 2017;102(5):416-426.
288. Robinson TN, Killen JD, Kraemer HC, et al. Dance and reducing television viewing to prevent weight gain in African-American girls: the Stanford GEMS pilot study. *Ethn Dis.* 2003;13:S65-77.
289. Robinson TN, Matheson DM, Kraemer HC, et al. A randomized controlled trial of culturally tailored dance and reducing screen time to prevent weight gain in low-income African American girls: Stanford GEMS. *Arch Pediatr Adolesc Med.* 2010;164:995-1004.
290. Robinson TN, Kraemer HC, Matheson DM, et al. Stanford GEMS phase 2 obesity prevention trial for low-income African-American girls: design and sample baseline characteristics. *Contemp Clin Trials.* 2008;29:56-69.
291. Rodearmel SJ, Wyatt HR, Barry MJ, et al. A family-based approach to preventing excessive weight gain. *Obesity (Silver Spring).* 2006;14:1392-1401.

292. Rodearmel SJ, Wyatt HR, Stroebele N, Smith SM, Ogden LG, Hill JO. Small changes in dietary sugar and physical activity as an approach to preventing excessive weight gain: the America on the Move family study. *Pediatrics.* 2007;120:e869-879.
293. Holm K, Wyatt H, Murphy J, Hill J, Odgen L. Parental influence on child change in physical activity during a family-based intervention for child weight gain prevention. *J Phys Act Health.* 2012;9:661-669.
294. Roemmich JN, Gurgol CM, Epstein LH. Open-loop feedback increases physical activity of youth. *Med Sci Sports Exerc.* 2004;36:668-673.
295. Rooney BL, Gritt LR, Havens SJ, Mathiason MA, Clough EA. Growing healthy families: family use of pedometers to increase physical activity and slow the rate of obesity. *WMJ.* 2005;104:54-60.
296. Rosado JL, del R, Arellano, M, Montemayor K, Garcia OP, Caamano M, C. An increase of cereal intake as an approach to weight reduction in children is effective only when accompanied by nutrition education: a randomized controlled trial. *Nutr J.* 2008;7:28.
297. Rosenkranz RR, Behrens TK, Dzewaltowski DA. A group-randomized controlled trial for health promotion in Girl Scouts: healthier troops in a SNAP (Scouting Nutrition & Activity Program). *BMC Public Health.* 2010;10:81.
298. Ruotsalainen H, Kyngas H, Tammelin T, Heikkinen H, Kaariainen M. Effectiveness of Facebook-Delivered Lifestyle Counselling and Physical Activity Self-Monitoring on Physical Activity and Body Mass Index in Overweight and Obese Adolescents: A Randomized Controlled Trial. *Nursing Research and Practice.* 2015.
299. Sacher PM, Kolotourou M, Chadwick PM, et al. Randomized controlled trial of the MEND program: a family-based community intervention for childhood obesity. *Obesity (Silver Spring).* 2010;18 Suppl 1:S62-68.
300. Saelens BE, Sallis JF, Wilfley DE, Patrick K, Cella JA, Buchta R. Behavioral weight control for overweight adolescents initiated in primary care. *Obes Res.* 2002;10:22-32.
301. Saelens BE, Grow HM, Stark LJ, Seeley RJ, Roehrig H. Efficacy of increasing physical activity to reduce children's visceral fat: a pilot randomized controlled trial. *Int J Pediatr Obes.* 2011;6:102-112.
302. Saelens BE, Lozano P, Scholz K. A randomized clinical trial comparing delivery of behavioral pediatric obesity treatment using standard and enhanced motivational approaches. *J Pediatr Psychol.* 2013;38:954-964.
303. Savoye M, Shaw M, Dziura J, et al. Effects of a weight management program on body composition and metabolic parameters in overweight children: a randomized controlled trial. *JAMA.* 2007;297:2697-2704.
304. Savoye M, Nowicka P, Shaw M, et al. Long-term results of an obesity program in an ethnically diverse pediatric population. *Pediatrics.* 2011;127:402-410.
305. Shaw M, Savoye M, Cali A, Dziura J, Tamborlane WV, Caprio S. Effect of a successful intensive lifestyle program on insulin sensitivity and glucose tolerance in obese youth. *Diabetes Care.* 2009;32:45-47.
306. Savoye M, Caprio S, Dziura J, et al. Reversal of early abnormalities in glucose metabolism in obese youth: results of an intensive lifestyle randomized controlled trial. *Diabetes Care.* 2014;37:317-324.
307. Serra-Paya N, Ensenyat A, Real J, et al. Evaluation of a family intervention programme for the treatment of overweight and obese children (Nereu Programme): a randomized clinical trial study protocol. *BMC Public Health.* 2013;13:1000.
308. Serra-Paya N, Ensenyat A, Castro-Vinuales I, et al. Effectiveness of a Multi-Component Intervention for Overweight and Obese Children (Nereu Program): A Randomized Controlled Trial. *PLoS One.* 2015;10(12):e0144502.
309. Sharifah WW, Nur HH, Ruzita AT, Roslee R, Reilly JJ. The Malaysian Childhood Obesity Treatment Trial (MASCOT). *Malays J Nutr.* 2011;17:229-236.
310. Wafa SW, Talib RA, Hamzaid NH, et al. Randomized controlled trial of a good practice approach to treatment of childhood obesity in Malaysia: Malaysian Childhood Obesity Treatment Trial (MASCOT). *Int J Pediatr Obes.* 2011;6:e62-69.
311. Shelton D, Le G, K, Norton L, Stanton-Cook S, Morgan J, Masterman P. Randomised controlled trial: A parent-based group education programme for overweight children. *J Paediatr Child Health.* 2007;43:799-805.
312. Sherwood NE, JaKa MM, Crain AL, Martinson BC, Hayes MG, Anderson JD. Pediatric Primary Care-Based Obesity Prevention for Parents of Preschool Children: A Pilot Study. *Childhood Obesity.* 2015;11(6):674-682.

313. Nguyen B, Shrewsbury VA, O'Connor J, et al. Two-year outcomes of an adjunctive telephone coaching and electronic contact intervention for adolescent weight-loss maintenance: the Loozit randomized controlled trial. *Int J Obes (Lond).* 2013;37:468-472.
314. Nguyen B, McGregor KA, O'Connor J, et al. Recruitment challenges and recommendations for adolescent obesity trials. *J Paediatr Child Health.* 2012;48:38-43.
315. Nguyen B, Shrewsbury VA, O'Connor J, et al. Twelve-month outcomes of the loozit randomized controlled trial: a community-based healthy lifestyle program for overweight and obese adolescents. *Arch Pediatr Adolesc Med.* 2012;166:170-177.
316. Kornman KP, Shrewsbury VA, Chou AC, et al. Electronic therapeutic contact for adolescent weight management: the Loozit study. *Telemed J E Health.* 2010;16:678-685.
317. Shrewsbury VA, Nguyen B, O'Connor J, et al. Short-term outcomes of community-based adolescent weight management: The Loozit® Study. *BMC Pediatr.* 2011;11:13.
318. Shrewsbury VA, O'Connor J, Steinbeck KS, et al. A randomised controlled trial of a community-based healthy lifestyle program for overweight and obese adolescents: the Loozit study protocol. *BMC Public Health.* 2009;9:119.
319. Siwik V, Kutob R, Ritenbaugh C, et al. Intervention in overweight children improves body mass index (BMI) and physical activity. *J Am Board Fam Med.* 2013;26:126-137.
320. Skouteris H, McCabe M, Swinburn B, Hill B. Healthy eating and obesity prevention for preschoolers: a randomised controlled trial. *BMC Public Health.* 2010;10:220.
321. Skouteris H, Hill B, McCabe M, Swinburn B, Busija L. A parent-based intervention to promote healthy eating and active behaviours in pre-school children: evaluation of the MEND 2–4 randomized controlled trial. *Pediatr Obes.* 2016;11(1):4-10.
322. Slusser W, Frankel F, Robison K, Fischer H, Cumberland WG, Neumann C. Pediatric overweight prevention through a parent training program for 2-4 year old Latino children. *Child Obes.* 2012;8:52-59.
323. Small L, Bonds-McClain D, Melnyk B, Vaughan L, Gannon AM. The Preliminary Effects of a Primary Care-Based Randomized Treatment Trial With Overweight and Obese Young Children and Their Parents. *J Pediatr Health Care.* 2013;28:198-207.
324. Kuhl ES, Clifford LM, Bandstra NF, et al. Examination of the association between lifestyle behavior changes and weight outcomes in preschoolers receiving treatment for obesity. *Health Psychol.* 2014;33:95-98.
325. Stark LJ, Spear S, Boles R, et al. A pilot randomized controlled trial of a clinic and home-based behavioral intervention to decrease obesity in preschoolers. *Obesity (Silver Spring).* 2011;19:134-141.
326. Jensen CD, Aylward BS, Steele RG. Predictors of attendance in a practical clinical trial of two pediatric weight management interventions. *Obesity (Silver Spring).* 2012;20:2250-2256.
327. Steele RG, Aylward BS, Jensen CD, Cushing CC, Davis AM, Bovaird JA. Comparison of a family-based group intervention for youths with obesity to a brief individual family intervention: a practical clinical trial of positively fit. *J Pediatr Psychol.* 2012;37:53-63.
328. Stettler N, Wrotniak BH, Hill DL, et al. Prevention of excess weight gain in paediatric primary care: beverages only or multiple lifestyle factors. The Smart Step Study, a cluster-randomized clinical trial. *Pediatr Obes.* 2015;10(4):267-274.
329. Hughes AR, Stewart L, Chapple J, et al. Randomized, controlled trial of a best-practice individualized behavioral program for treatment of childhood overweight: Scottish Childhood Overweight Treatment Trial (SCOTT). *Pediatrics.* 2008;121:e539-546.
330. Stewart L, Houghton J, Hughes AR, Pearson D, Reilly JJ. Dietetic management of pediatric overweight: development and description of a practical and evidence-based behavioral approach. *J Am Diet Assoc.* 2005;105:1810-1815.
331. Stolley MR, Fitzgibbon ML. Effects of an obesity prevention program on the eating behavior of African American mothers and daughters. *Health Educ Behav.* 1997;24:152-164.
332. Story M, Sherwood NE, Himes JH, et al. An after-school obesity prevention program for African-American girls: the Minnesota GEMS pilot study. *Ethn Dis.* 2003;13:S54-64.

333. Story M, Hannan PJ, Fulkerson JA, et al. Bright Start: Description and main outcomes from a group-randomized obesity prevention trial in American Indian children. *Obesity (Silver Spring).* 2012;20:2241-2249.
334. Arcan C, Hannan P, Himes J, et al. Intervention Effects on Kindergarten and First-Grade Teachers' Classroom Food Practices and Food-Related Beliefs in American Indian Reservation Schools. *J Acad Nutr Diet.* 2013;113:1076-1083.
335. Stovitz SD, Berge JM, Wetzsteon RJ, Sherwood NE, Hannan PJ, Himes JH. Stage 1 treatment of pediatric overweight and obesity: a pilot and feasibility randomized controlled trial. *Child Obes.* 2014;10:50-57.
336. Tanofsky-Kraff M, Shomaker LB, Wilfley DE, et al. Excess Weight Gain Prevention in Adolescents: Three-Year Outcome Following a Randomized Controlled Trial. *J Consult Clin Psychol.* 2017;85(3):218-227.
337. Tanofsky-Kraff M, Shomaker LB, Wilfley DE, et al. Targeted prevention of excess weight gain and eating disorders in high-risk adolescent girls: a randomized controlled trial. *American Journal of Clinical Nutrition.* 2014;100(4):1010-1018.
338. Tanofsky-Kraff M, Wilfley DE, Young JF, et al. A pilot study of interpersonal psychotherapy for preventing excess weight gain in adolescent girls at-risk for obesity. *Int J Eat Disord* 2010;43:701-706.
339. Woo B, J A, Price SN, Gonzalez-Suarez E, et al. Parental perceptions of a motivational interviewing-based pediatric obesity prevention intervention. *Clin Pediatr (Phila).* 2013;52:540-548.
340. Wright DR, Taveras EM, Gillman MW, et al. The cost of a primary care-based childhood obesity prevention intervention. *BMC Health Serv Res.* 2014;14:44.
341. Taveras EM, Gortmaker SL, Hohman KH, et al. Randomized controlled trial to improve primary care to prevent and manage childhood obesity: the High Five for Kids study. *Arch Pediatr Adolesc Med.* 2011;165:714-722.
342. Rifas-Shiman SL, Taveras EM, Gortmaker SL, et al. Two-year follow-up of a primary care-based intervention to prevent and manage childhood obesity: the High Five for Kids study. *Pediatric obesity.* 2017;12(3):e24-e27.
343. Taveras EM, McDonald J, O'Brien A, et al. Healthy Habits, Happy Homes: methods and baseline data of a randomized controlled trial to improve household routines for obesity prevention. *Prev Med.* 2012;55:418-426.
344. Haines J, McDonald J, O'Brien A, et al. Healthy habits, happy homes: randomized trial to improve household routines for obesity prevention among preschool-aged children. *JAMA Pediatr.* 2013;167(11):1072-1079.
345. Taveras EM, Marshall R, Horan CM, et al. Rationale and design of the STAR randomized controlled trial to accelerate adoption of childhood obesity comparative effectiveness research. *Contemp Clin Trials.* 2013;34:101-108.
346. Taveras EM, Marshall R, Kleinman KP, et al. Comparative effectiveness of childhood obesity interventions in pediatric primary care: a cluster-randomized clinical trial. *JAMA Pediatr.* 2015;169(6):535-542.
347. Taveras EM, Marshall R, Sharifi M, et al. Comparative Effectiveness of Clinical-Community Childhood Obesity Interventions A Randomized Clinical Trial. *JAMA Pediatr.* 2017;171(8).
348. Taveras EM, Marshall R, Sharifi M, et al. Connect for Health: Design of a clinical-community childhood obesity intervention testing best practices of positive outliers. *Contemp Clin Trials.* 2015;45:287-295.
349. Taylor RW, Brown D, Dawson AM, et al. Motivational interviewing for screening and feedback and encouraging lifestyle changes to reduce relative weight in 4-8 year old children: design of the MInT study. *BMC Public Health.* 2010;10:271.
350. Taylor RW, Cox A, Knight L, et al. A tailored family-based obesity intervention: a randomized trial. *Pediatrics.* 2015;136(2):281-289.
351. Tjønna AE, Stølen TO, Bye A, et al. Aerobic interval training reduces cardiovascular risk factors more than a multitreatment approach in overweight adolescents. *Clin Sci (Lond).* 2009;116:317-326.
352. Adams AK, LaRowe TL, Cronin KA, et al. The Healthy Children, Strong Families intervention: design and community participation. *J Prim Prev.* 2012;33(4):175-185.
353. Tomayko EJ, Prince RJ, Cronin KA, Adams AK. The Healthy Children, Strong Families intervention promotes improvements in nutrition, activity and body weight in American Indian families with young children. *Public health nutrition.* 2016;19(15):2850-2859.

354. Toulabi T, Khosh N, Nikoo, M, Amini F, Nazari H, Mardani M. The influence of a behavior modification interventional program on body mass index in obese adolescents. *J Formos Med Assoc.* 2012;111:153-159.
355. Trost SG, Sundal D, Foster GD, Lent MR, Vojta D. Effects of a pediatric weight management program with and without active video games a randomized trial. *JAMA pediatrics.* 2014;168(5):407-413.
356. Tsiros MD, Sinn N, Brennan L, et al. Cognitive behavioral therapy improves diet and body composition in overweight and obese adolescents. *Am J Clin Nutr.* 2008;87:1134-1140.
357. van Grieken A, Veldhuis L, Renders CM, et al. Population-based childhood overweight prevention: outcomes of the 'Be active, eat right' study. *PLoS One.* 2013;8:e65376.
358. Veldhuis L, Struijk MK, Kroeze W, et al. Be active, eat right', evaluation of an overweight prevention protocol among 5-year-old children: design of a cluster randomised controlled trial. *BMC Public Health.* 2009;9:177.
359. van Grieken A, Renders CM, Veldhuis L, Looman CW, Hirasing RA, Raat H. Promotion of a healthy lifestyle among 5-year-old overweight children: health behavior outcomes of the 'Be active, eat right' study. *BMC Public Health.* 2014;14:59.
360. Vos RC, Pijl H, Wit JM, van Z, E W, van d, Bent, C, Houdijk EC. The effect of multidisciplinary lifestyle intervention on the pre- and postprandial plasma gut Peptide concentrations in children with obesity. *ISRN Endocrinol.* 2011;2011:353756.
361. Vos RC, Huisman SD, Houdijk EC, Pijl H, Wit JM. The effect of family-based multidisciplinary cognitive behavioral treatment on health-related quality of life in childhood obesity. *Qual Life Res.* 2012;21:1587-1594.
362. Vos RC, Wit JM, Pijl H, Houdijk EC. Long-term effect of lifestyle intervention on adiposity, metabolic parameters, inflammation and physical fitness in obese children: a randomized controlled trial. *Nutr Diabetes.* 2011;1:e9.
363. Vos RC, Wit JM, Pijl H, Kruyff CC, Houdijk EC. The effect of family-based multidisciplinary cognitive behavioral treatment in children with obesity: study protocol for a randomized controlled trial. *Trials.* 2011;12:110.
364. Wadden TA, Stunkard AJ, Rich L, Rubin CJ, Sweidel G, McKinney S. Obesity in black adolescent girls: a controlled clinical trial of treatment by diet, behavior modification, and parental support. *Pediatrics.* 1990;85:345-352.
365. Wake M, Baur LA, Gerner B, et al. Outcomes and costs of primary care surveillance and intervention for overweight or obese children: the LEAP 2 randomised controlled trial. *BMJ.* 2009;339:b3308.
366. Wake M, Lycett K, Clifford SA, et al. Shared care obesity management in 3-10 year old children: 12 month outcomes of HopSCOTCH randomised trial. *BMJ.* 2013;346:f3092.
367. Wake M, Lycett K, Sabin MA, et al. A shared-care model of obesity treatment for 3-10 year old children: protocol for the HopSCOTCH randomised controlled trial. *BMC Pediatr.* 2012;12:39.
368. Waling M, Bäcklund C, Lind T, Larsson C. Effects on metabolic health after a 1-year-lifestyle intervention in overweight and obese children: a randomized controlled trial. *J Nutr Metab.* 2012;2012:913965.
369. Waling M, Lind T, Hernell O, Larsson C. A one-year intervention has modest effects on energy and macronutrient intakes of overweight and obese Swedish children. *J Nutr.* 2010;140:1793-1798.
370. Bäcklund C, Sundelin G, Larsson C. Effect of a 1-year lifestyle intervention on physical activity in overweight and obese children. *Adv Physiother.* 2011;13:87-96.
371. Bäcklund C, Sundelin G, Larsson C. Effects of a 2-year lifestyle intervention on physical activity in overweight and obese children. *Adv Physiother.* 2011;13:97-109.
372. Waling M, Larsson C. Improved dietary intake among overweight and obese children followed from 8 to 12 years of age in a randomised controlled trial. *J Nutr Sci* 2012;1:1-11.
373. Walpole B, Dettmer E, Morrongiello BA, McCrindle BW, Hamilton J. Motivational interviewing to enhance self-efficacy and promote weight loss in overweight and obese adolescents: a randomized controlled trial. *J Pediatr Psychol.* 2013;38:944-953.
374. Walton K, Filion AJ, Gross D, et al. Parents and Tots Together: Pilot randomized controlled trial of a family-based obesity prevention intervention in Canada. *Canadian Journal of Public Health-Revue Canadienne De Sante Publique.* 2015;106(8):E555-E562.

375. Warren JM, Henry CJ, Lightowler HJ, Bradshaw SM, Perwaiz S. Evaluation of a pilot school programme aimed at the prevention of obesity in children. *Health Promot Int.* 2003;18:287-296.
376. Weigel C, Kokocinski K, Lederer P, Dotsch J, Rascher W, Knerr I. Childhood obesity: concept, feasibility, and interim results of a local group-based, long-term treatment program. *J Nutr Educ Behav.* 2008;40:369-373.
377. Weigensberg MJ, Lane CJ, Ávila Q, et al. Imagine HEALTH: results from a randomized pilot lifestyle intervention for obese Latino adolescents using Interactive Guided ImagerySM. *BMC Complement Altern Med.* 2014;14:28.
378. Weintraub DL, Tirumalai EC, Haydel KF, Fujimoto M, Fulton JE, Robinson TN. Team sports for overweight children: the Stanford Sports to Prevent Obesity Randomized Trial (SPORT). *Arch Pediatr Adolesc Med.* 2008;162:232-237.
379. Wengle JG, Hamilton JK, Manlhiot C, et al. The 'Golden Keys' to health - a healthy lifestyle intervention with randomized individual mentorship for overweight and obesity in adolescents. *Paediatr Child Health.* 2011;16:473-478.
380. West F, Sanders MR, Cleghorn GJ, Davies PS. Randomised clinical trial of a family-based lifestyle intervention for childhood obesity involving parents as the exclusive agents of change. *Behav Res Ther.* 2010;48:1170-1179.
381. Williamson DA, Walden HM, White MA, et al. Two-year internet-based randomized controlled trial for weight loss in African-American girls. *Obesity (Silver Spring).* 2006;14:1231-1243.
382. Williamson DA, Martin PD, White MA, et al. Efficacy of an internet-based behavioral weight loss program for overweight adolescent African-American girls. *Eat Weight Disord.* 2005;10:193-203.
383. White MA, Martin PD, Newton RL, et al. Mediators of weight loss in a family-based intervention presented over the internet. *Obes Res.* 2004;12:1050-1059.
384. Theim KR, Sinton MM, Stein RI, et al. Preadolescents' and parents' dietary coping efficacy during behavioral family-based weight control treatment. *J Youth Adolesc.* 2012;41:86-97.
385. Wilfley DE, Stein RI, Saelens BE, et al. Efficacy of maintenance treatment approaches for childhood overweight: a randomized controlled trial. *JAMA.* 2007;298:1661-1673.
386. Goldschmidt AB, Stein RI, Saelens BE, Theim KR, Epstein LH, Wilfley DE. Importance of early weight change in a pediatric weight management trial. *Pediatrics.* 2011;128:e33-39.
387. Theim KR, Sinton MM, Goldschmidt AB, et al. Adherence to behavioral targets and treatment attendance during a pediatric weight control trial. *Obesity (Silver Spring).* 2013;21:394-397.
388. Willeboordse M, van de Kant KDG, Tan FES, et al. A Multifactorial Weight Reduction Programme for Children with Overweight and Asthma: A Randomized Controlled Trial. *PLoS One.* 2016;11(6).
389. Willeboordse M, van de Kant KDG, de Laat MN, van Schayck OCP, Mulkens S, Dompeling E. Multifactorial intervention for children with asthma and overweight (Mikado): study design of a randomised controlled trial. *BMC Public Health.* 2013;13.
390. Wright K, Norris K, Newman G, J, Suro Z. Improving healthy dietary behaviors, nutrition knowledge, and self-efficacy among underserved school children with parent and community involvement. *Child Obes.* 2012;8:347-356.
391. Wright K, Giger JN, Norris K, Suro Z. Impact of a nurse-directed, coordinated school health program to enhance physical activity behaviors and reduce body mass index among minority children: a parallel-group, randomized control trial. *Int J Nurs Stud.* 2013;50:727-737.
392. Wright K, Suro Z. Using Community–Academic Partnerships and a Comprehensive School-Based Program to Decrease Health Disparities in Activity in School-Aged Children. *J Prev Interv Community.* 2014;42(2):125-139.
393. Wright JA, Phillips BD, Watson BL, Newby PK, Norman GJ, Adams WG. Randomized trial of a family-based, automated, conversational obesity treatment program for underserved populations. *Obesity (Silver Spring).* 2013;21:E369-E378.
394. Yackobovitch-Gavan M, Nagelberg N, Phillip M, Ashkenazi-Hoffnung L, Hershkovitz E, Shalitin S. The influence of diet and/or exercise and parental compliance on health-related quality of life in obese children. *Nutr Res.* 2009;29:397-404.

395. Shalitin S, Ashkenazi-Hoffnung L, Yackobovitch-Gavan M, et al. Effects of a twelve-week randomized intervention of exercise and/or diet on weight loss and weight maintenance, and other metabolic parameters in obese preadolescent children. *Horm Res* 2009;72(5):287-301.
396. Yilmaz G, Caylan ND, Karacan CD. An intervention to preschool children for reducing screen time: a randomized controlled trial. *Child Care Hlth Dev.* 2015;41(3):443-449.
397. Yin TJ, Wu FL, Liu YL, Yu S. Effects of a weight-loss program for obese children: a mix of attributes' approach.'. *J Nurs Res.* 2005;13:21-30.
398. Yin Z, Moore JB, Johnson MH, Vernon MM, Gutin B. The impact of a 3-year after-school obesity prevention program in elementary school children. *Child Obes.* 2012;8:60-70.
399. Gutin B, Yin Z, Johnson M, Barbeau P. Preliminary findings of the effect of a 3-year after-school physical activity intervention on fitness and body fat: the Medical College of Georgia Fitkid Project. *Int J Pediatr Obes.* 2008;3 Suppl 1:3-9.
400. Wang LY, Gutin B, Barbeau P, et al. Cost-effectiveness of a school-based obesity prevention program. *J Sch Health.* 2008;78:619-624.
401. Yin Z, Gutin B, Johnson MH, et al. An environmental approach to obesity prevention in children: Medical College of Georgia FitKid Project year 1 results. *Obes Res.* 2005;13:2153-2161.
402. Yin Z, Hanes J, Jr, Moore JB, Humbles P, Barbeau P, Gutin B. An after-school physical activity program for obesity prevention in children: the Medical College of Georgia FitKid Project. *Eval Health Prof.* 2005;28:67-89.
403. Yin Z, Moore JB, Johnson MH, et al. The Medical College of Georgia FitKid Project: the relations between program attendance and changes in outcomes in year 1. *Int J Obes (Lond).* 2005;29:S40S45.
